# Development of the International Federation for Surgery of Obesity and Metabolic Disorders-European Chapter (IFSO-EC) Grade-Based Guidelines on the Surgical Treatment of Obesity Using Multimodal Strategies: Design and Methodological Aspects

**DOI:** 10.3390/jcm13175106

**Published:** 2024-08-28

**Authors:** Maurizio De Luca, Amanda Belluzzi, Paulina Salminen, Marco Bueter, Juan Pujol-Rafols, Nasser Sakran, Christine Stier, Halit Eren Taskin, Sonja Chiappetta, Francesco Maria Carrano, Nicola Di Lorenzo, Simon Nienhuijs, Ramón Vilallonga Puy, Erik Stenberg, Marloes Emous, Gerhard Prager, Jacques Himpens, Daniel Moritz Felsenreich, Antonio Iannelli, Chetan Parmar, Catalin Copaescu, Martin Fried, Elena Ruiz-Úcar, Ricardo V. Cohen, Stefano Olmi, Luigi Angrisani, Rui Ribeiro, Giulia Bandini, Daniele Scoccimarro, Benedetta Ragghianti, Matteo Monami

**Affiliations:** 1 Department of General, Emergency and Metabolic Surgery, Rovigo Hospital, 45100 Rovigo, Italy; amandabelluzzi@gmail.com; 2 Department of Surgery, University of Turku, 20014 Turku, Finland; paulina.salminen@tyks.fi; 3 Department of Surgery and Transplantation, University Hospital Zurich, University of Zurich, 8006 Zurich, Switzerland; m.bueter@spitalmaennedorf.ch; 4 HM Delfos, 08023 Barcelona, Spain; juan.pujol.rafols@gmail.com; 5 Department of General Surgery, Holy Family Hospital, Nazareth 1601001, Israel; sakranas@gmail.com; 6 The Azrieli Faculty of Medicine, Bar-Ilan University, Ramat Gan 5290002, Israel; 7 Department of Surgery, Bariatric Endoscopy, Medical Faculty Mannheim, Heidelberg University, 69167 Heidelberg, Germany; christinestier@gmail.com; 8 Department of Surgery, Cerrahpasa Medical Faculty, Istanbul University, 34320 Istanbul, Turkey; eren_taskin@hotmail.com; 9 Bariatric and Metabolic Surgery Unit, Department of General and Laparoscopic Surgery, Ospedale Evangelico Betania, 80147 Naples, Italy; drschiappetta@gmail.com; 10 Department of Medical Surgical Sciences and Translational Medicine, Sant’Andrea Hospital, Sapienza University, 00189 Rome, Italy; carranofm@gmail.com; 11 Department of Surgical Sciences, University of Tor Vergata, 00133 Rome, Italy; nicoladilorenzo@me.com; 12 Department of Surgery and Gastroenterology, Catharina Hospital, 5623 Eindhoven, The Netherlands; simon.nienhuijs@catharinaziekenhuis.nl; 13 Endocrine-Metabolic and Bariatric Surgery Unit, Vall Hebron Barcelona Hospital Campus, Universitat Autònoma de Barcelona, 08035 Barcelona, Spain; ramon.vilallonga@vallhebron.cat; 14 Department of Surgery, Faculty of Medicine and Health, Örebro University, 70182 Örebro, Sweden; erik.stenberg@regionorebrolan.se; 15 Center for Obesity Northern-Netherlands (CON), Department of Bariatric and Metabolic Surgery, Medical Center, 8934 Leeuwarden, The Netherlands; marloes.emous@mcl.nl; 16 Division of Visceral Surgery, Department of General Surgery, Medical University of Vienna, 1090 Vienna, Austria; gerhard.prager@meduniwien.ac.at (G.P.); moritz.felsenreich@meduniwien.ac.at (D.M.F.); 17 Delta CHIREC Hospital (Belgian Registry), 1160 Brussels, Belgium; jacques_himpens@hotmail.com; 18 Digestive Surgery and Liver Transplantation Unit, Université Côte d’Azur, 06103 Nice, France; iannelli.a@chu-nice.fr; 19 Department of Surgery, Whittington Hospital, University College, London N19 5NF, UK; cparmar@nhs.net; 20 Department of Surgery, Ponderas Academic Hospital, 014142 Bucharest, Romania; catalincopaescu@gmail.com; 21 OB Klinika—Center for Treatment of Obesity and Metabolic Disorders, 1300 Prague, Czech Republic; docfried@volny.cz; 22 General and Digestive Surgery Department, Fuenlabrada University Hospital, Rey Juan Carlos University, 28942 Madrid, Spain; eruizucar@gmail.com; 23 The Center for Obesity and Diabetes, Hospital Alemao Oswaldo Cruz, Sao Paulo 01323-20, Brazil; rvcohen@mac.com; 24 San Marco Hospital GSD, 24046 Zingonia, Italy; stefano.olmi@gmail.com; 25 Public Health Department, School of Medicine, University Federico II of Naples, 80138 Naples, Italy; luigiangrisani@chirurgiaobesita.it; 26 Centro Multidisciplinar Do Tratamento da Obesidade, Hospital Lusíadas Amadora e Lisboa, 2724-002 Amadora, Portugal; ruijsribeiro@gmail.com; 27 Diabetic Foot Unit, University of Florence and AOU-Careggi, 50121 Florence, Italy; giulia.bandini@unifi.it (G.B.); daniele.scoccimarro@unifi.it (D.S.); benedetta.ragghianti@unifi.it (B.R.); matteo.monami@unifi.it (M.M.)

**Keywords:** obesity, metabolic bariatric surgery, obesity-related medical conditions, obesity management medications, lifestyle interventions, endoscopic weight loss procedures, guidelines

## Abstract

**Background**: The prevalence of obesity is already a worldwide health concern. The development of straightforward guidelines regarding the whole available armamentarium (i.e., medical, endoscopic, and surgical interventions in conjunction with a guidance program) is paramount to offering the best multimodal approach to patients with obesity. **Methods**: The International Federation for Surgery of Obesity and Metabolic Disorders-European Chapter (IFSO-EC) identified a panel of experts to develop the present guidelines. The panel formulated a series of clinical questions (based on the patient, intervention, comparison, and outcome conceptual framework), which have been voted on and approved. A GRADE methodology will be applied to assess the quality of evidence and formulate recommendations employed to minimize selection and information biases. This approach aims to enhance the reliability and validity of recommendations, promoting greater adherence to the best available evidence. **Results**: These guidelines are intended for adult patients with a body mass index (BMI) ≥ 30 kg/m^2^ who are candidates for metabolic bariatric surgery (MBS). The expert panel responsible for developing these guidelines comprised 25 panelists (92% were bariatric surgeons) and 3 evidence reviewers, with an average age of 50.1 ± 10.2 years. The panel focused on 3 key questions regarding the combined use of structured lifestyle interventions, approved obesity management medications, and endoscopic weight loss procedures with MBS. **Conclusions**: The complexity of obesity as a chronic disease requires a comprehensive knowledge of all the available and feasible therapeutic options. The IFSO-EC society felt the urgent need to develop methodologically valid guidelines to give a full picture and awareness of the possible surgical and non-surgical therapeutic strategies employed with a multimodal approach.

## 1. Introduction

Obesity is a chronic disease that has become a major public health concern worldwide. This condition affects individuals of all ages and backgrounds, leading to significant health, social, and economic consequences. The primary cause of obesity is an energy imbalance between calories consumed and calories expended. This imbalance can result from various factors, including dietary habits, physical inactivity, sedentary lifestyles, genetic predisposition, environmental (e.g., urbanization, lack of access to healthy foods, etc.) and psychological (e.g., stress, depression, and anxiety) factors [1].

Obesity is associated with a high number of health risks and complications, including cardiovascular and metabolic diseases. Obesity has been demonstrated to be capable of increasing the risk of ischemic heart disease, hypertension, and stroke due to the strain excess weight places on the heart and blood vessels [2]. Obesity is one of the most important risk factors for type 2 diabetes. Visceral body fat can lead to insulin resistance, a well-known precursor of type 2 diabetes [3]. Notably, overweight and obesity are also associated with other conditions such as musculoskeletal disorders (e.g., osteoarthritis), more common in obese individuals due to the additional stress on joints and tendons [2], some types of malignancies, including breast, colon, and endometrial cancers, and respiratory disease (e.g., obstructive sleep apnea) [4,5].

Preventing and managing obesity requires a multifaceted approach. Lifestyle, pharmacological, and surgical interventions have been developed to address the complex nature of this disease and its related medical conditions [5,6,7,8,9]. The first step should always be represented by lifestyle changes. Patients affected by overweight or obesity should be encouraged to adopt a balanced diet rich in fruits, vegetables, whole grains, and lean proteins while limiting the intake of processed and high-calorie foods. Promoting regular physical activity, such as walking, cycling, and other forms of exercise, should be a cornerstone of any therapeutic approach to help maintain a healthy weight [9].

When lifestyle changes are inadequate to achieve or maintain weight loss achievements or to prevent weight recurrence, pharmacological interventions could be a possible additional strategy, including FDA and EMA-approved drugs such as orlistat, lorcaserin, phentermine/topiramate, naltrexone/bupropion, liraglutide, semaglutide, and tirzepatide [10]. These drugs are capable of reducing food intake and/or increasing satiety, thereby promoting weight loss. There is an impressive number of obesity management medications under investigation, such as GLP1/glucagon dual agonists, GIP/GLP1 dual agonists, GIP/GLP1/glucagon tri-agonists, leptin sensitizers, etc. [9].

Several patients, particularly those affected by the previously “morbid obesity”, often require a surgical intervention (i.e., metabolic and bariatric surgery—MBS), such as Sleeve Gastrectomy (SG), Roux-en-Y Gastric Bypass (RYGB), One Anastomosis Gastric Bypass (OAGB), Adjustable Gastric Banding (AGB), and Single Anastomosis Duodeno—Ileal Bypass with Sleeve (SADI-S). These procedures lead to significant and sustained weight loss by modifying the anatomy of the gastrointestinal tract and different absorption of micro and macronutrients. Another possible non-surgical interventional strategy could be represented by endoscopic procedures, such as IntraGastric Balloons (IGB) and Endoscopic Sleeve Gastroplasty (ESG), which are minimally invasive options with promising results in terms of weight loss and obesity-related medical condition remission [5,6,7,8,9,11]. However, weight loss is not the only target of anti-obesity strategies. Glucometabolic endpoints, remission of obesity-related medical conditions, and quality of life should also be considered among critical outcomes when rating and selecting different weight loss strategies [12,13,14,15,16,17,18].

Despite all physicians involved in the surgical treatment of obesity being aware (and entirely convinced) about the importance of “non-surgical management” of patients undergoing MBS, the concurrent adoption of lifestyle programs and/or anti-obesity drugs is often neglected. As an example, by reviewing a recent meta-analysis collecting randomized control trials (RCT) on MBS, only a small fraction of the included studies adopted intensive lifestyle programs as add-on therapy to MBS [7,15,16,17,19,20,21]. Moreover, even fewer studies have explored the effectiveness of combined treatments (surgical + nonsurgical) compared to surgical interventions alone [11,22,23,24,25].

The present methodological article aims to transparently pre-specify the entire process behind the development of this guideline promoted by the International Federation for Surgery of Obesity and Metabolic Disorders-European Chapter (IFSO-EC) scientific society. The IFSO-EC society in fact recognized the need for reinforcing the combined adoption of nonsurgical strategies with surgical procedures and decided to develop a formal guideline aimed at making healthcare professionals aware of the possibility of a multimodal approach for the treatment of obesity and associated medical problems. In developing this guideline, the nominated panel of experts decided to use the Grading of Recommendations, Assessment, Development, and Evaluations (GRADE) methodology, which requires the identification of specific clinical questions and the definition of relevant outcomes for each one of those questions [25].

## 2. Materials and Methods

Characteristics of the Panel involved in the development of the guideline

Panel members, identified by the IFSO-EC (Table S1), elected a coordinator (MDL) and nominated the members of the Evidence Review Team (ERT). The latter actively collects and analyzes evidence without participating in the definition of clinical questions, outcomes, and recommendations. The complete list of the panel and ERT members, with their roles, is reported in Table S1. All panel members and the ERT compiled a declaration of potential conflicts of interest, which were collectively discussed to determine their relevance. In all cases, the reported conflicts were considered minimal or irrelevant. Therefore, all components of the panel and the ERT participated in elaborating all recommendations.

The panel of experts decided to follow the Grading of Recommendations, Assessment, Development, and Evaluation (GRADE) as described below. Moreover, they decided to base recommendations exclusively on the results of randomized clinical trials (RCT), planning several meta-analyses. The decision to exclude nonrandomized studies has been taken based on some methodological considerations. Nonrandomized studies, in fact, can introduce some bias, such as “selection bias”, often not present in RCTs. Selection bias is a critical concern in assessing the effectiveness of pharmacological and nonpharmacological interventions. Selection bias occurs when the allocation of participants to different intervention groups (or exposure categories) is not random. As a consequence, systematic differences in participant characteristics cannot completely be ruled out. These differences, rather than the intervention, could be responsible for between-group differences in the explored endpoints. The Cochrane Training website recommends avoiding the use of non-randomized studies in systematic reviews and meta-analyses, particularly when assessing an intervention, such as a surgical procedure. Nonrandomized studies should be considered only when “the question of interest cannot be answered by randomized trials”, as reported in Chapter 24 of the Cochrane manual [26,27].

GRADE methodology for the development of guidelines

The GRADE (Grading of Recommendations, Assessment, Development, and Evaluation) methodology is a systematic and transparent approach used to develop healthcare guidelines. By following this methodology, it is more likely to obtain recommendations based on the best available evidence and to achieve a more reliable balance between benefits and harms, patient values, and preferences [25]. The first step is the formulation of clear and focused clinical questions using the PICO format (population, intervention, comparator, outcomes). This step is crucial for the whole evidence review process. By clearly defining the clinical question, the panel of experts can ensure that the evidence gathered is relevant and specific to the healthcare decision-making. The next step is to identify, prioritize, and rate outcomes based on their importance to patients and decision-makers. Outcomes are classified as critical, important, or less important. This classification helps in focusing the evidence review on the most relevant outcomes, ensuring that the guidelines address the most significant aspects of patient care [25].

The GRADE methodology highlights the importance of systematic reviews in summarizing evidence. Systematic reviews involve a comprehensive search for relevant studies, followed by a critical appraisal and synthesis of the evidence. The summarized evidence is often presented in evidence profiles and tables summarizing findings, which provide a clear and concise overview of the available evidence for each investigated outcome. A pivotal component of the GRADE methodology is the assessment of the quality of evidence. This assessment relies on several factors, including: (i) Risk of bias: evaluating the methodological quality of the studies (see the paragraph below); (ii) Inconsistency: assessing the variability in study results; (iii) Indirectness: considering the applicability of the evidence to the clinical question; (iv) Imprecision: evaluating the certainty of the evidence; (v) Publication bias: checking for selective publication of studies. The quality of evidence is rated as high, moderate, low, or very low. This rating helps guideline developers to understanding to which level they can trust the evidence and informs the strength of the recommendations [25].

The panel of experts formulates recommendations based on the quality of evidence and the balance between benefits and harms. These recommendations should also consider patient values and preferences, resource use, and feasibility. The final step in the GRADE process is to grade the strength of the recommendations. Recommendations are classified as strong or weak. Strong recommendations indicate high confidence that the benefits outweigh the risks; on the contrary, weak recommendations suggest that the benefits and risks are closely balanced or uncertain [25].

Following the above-mentioned steps, for each question, the panel of experts nominated by the IFSO-EC society defined several clinical outcomes, potentially relevant for choosing different clinical options. Each outcome was then rated (from 1 to 9) for its importance. Those rating 7 or higher were classified as “critical” and represent the basis for developing the recommendation. Moreover, for each critical outcome (7 or higher), the evidence review team (ERT) will perform a systematic review (and meta-analyses whenever possible) of relevant studies, predefining search strategies and inclusion criteria, as stated in Table S2. Studies and related meta-analyses are assessed for methodological quality to verify the actual strength of available evidence.

Further assessments will include economic evaluations (usually based on cost-utility ratio, whenever possible), organizational impact, equity, acceptability, and feasibility. The final recommendation will include all those elements.

Evidence to Decision (EtD) framework

The EtD frameworks will be used to support the process of moving from evidence to decisions, trying to formulate an answer for each of the following criteria [27]:

- Is the problem a priority?
- How substantial are the desirable anticipated effects?
- How substantial are the anticipated undesirable effects?
- What is the overall certainty of the evidence of effects?
- Is there important uncertainty about how much people value the main outcomes?
- Does the balance between desirable effects and undesirable effects favor the option or the comparison?
- How large are the resource requirements (costs)?
- What is the certainty of the evidence of resource use?
- Does the cost-effectiveness of the option favor the option or the comparison?
- What would be the impact on health equity?
- Is the option acceptable to key stakeholders?
- Is the option feasible to implement?

The EtD frameworks serve as a structured and transparent approach to decision-making. These frameworks help panels of experts consider evidence when making decisions related to clinical recommendations, coverage choices, and health system or public health recommendations. By systematically evaluating relevant factors, such as anticipated effects, evidence quality, and feasibility, EtD frameworks ensure informed judgments and facilitate the dissemination of recommendations across different contexts [27].

Risk of Bias (RoB) assessment

When developing clinical guidelines, assessing the risk of bias (RoB) is crucial. RoB refers to the potential for systematic errors in research studies that could affect the validity of their findings [26]. We will assess the following risks for each included study, as reported in the RoB 2.0 checklist [28]:

- Study Design: Evaluate the study design. RCTs are generally considered less biased than observational studies, as specified above.
- Randomization and Allocation Concealment: Assess whether randomization was appropriately conducted and whether allocation was concealed.
- Blinding: Consider whether blinding (single-blind or double-blind) was implemented to minimize bias.
- Incomplete Outcome Data: Check if there were missing data and how they were handled.
- Selective Outcome Reporting: Ensure that all relevant outcomes were reported, not just positive or statistically significant ones.
- Funding Source: Investigate potential conflicts of interest related to funding sources.

DELPHI process

The definition of clinical questions (PICO) was performed using a two-step web-based Delphi method, a structured technique aimed at obtaining, by repeated rounds of questionnaires, a consensus opinion from a panel of experts in areas wherein evidence is scarce or conflicting [29]. The Delphi process is a structured communication technique originally developed as a systematic, interactive forecasting method that relies on a panel of experts. The Delphi process involves multiple rounds of questionnaires sent to a panel of selected experts. Each round is followed by a summary of the previous round’s results, which is shared with the panel [29]. The experts are encouraged to revise their earlier answers in light of the responses of other members of the panel. It is believed that during this process, the range of the answers will decrease and the group will converge towards the “correct” answer. The first step is to select a panel of experts who have relevant knowledge and experience regarding the topic of the questionnaire. The initial questionnaire is designed to gather the experts’ opinions on the topic. This round is usually open-ended to allow for a wide range of responses. The responses from the first round are collected and analyzed. Common themes and divergent opinions are identified. A second questionnaire is developed based on the analysis of the first round. This questionnaire is more structured and may include rating scales or multiple-choice questions to quantify the experts’ opinions. The process of collecting responses, analyzing them, and sending out revised questionnaires continues for several rounds. Each round provides feedback to the experts, allowing them to reconsider their views. The process continues until a consensus is reached or diminishing returns are observed. The outcome is a collective judgment of the panel, which is considered more reliable than individual opinions.

Between January and June 2024, panelists were invited to propose clinical questions with the PICO framework and to express their level of agreement or disagreement on each proposed question using a 5-point Likert scale (1, strongly disagree; 2, disagree; 3, agree; 4, mostly agree; and 5, strongly agree). Results will be expressed as a percentage of respondents who scored each item as 1 or 2 (disagreement) or as 3, 4, or 5 (agreement). A positive consensus was reached in cases of more than 66% agreement, a negative consensus in cases of more than 66% disagreement, and consensus was not reached when the sum for disagreement or agreement was below 66%. For the statements on which consensus had not been achieved, panelists were asked to re-rate in a second round their agreement/disagreement, after an internal discussion with all panelists. Only PICO achieving an agreement of more than 66% will be assessed using the GRADE methodology and subsequently included in the upcoming guideline.

## 3. Results

This guideline will apply to adult patients with body mass index (BMI) ≥ 30 kg/m^2^ requiring MBS. Healthcare systems, infrastructures, and human and financial resources across worldwide regions will be considered in developing these guidelines. The present guidelines will be used by healthcare professionals, including surgeons, Multi-Disciplinary Team (MDT) members, general practitioners, nutrition experts, psychologists, endocrinologists/diabetologists, and a patient representative.

The mean age of panelists (*n* = 25) and evidence reviewers (*n* = 3) was 50.1 ± 10.2 years, and the proportion of women was 32% (Table S1). The proportion of bariatric surgeons was 82%.

The panel proposed 3 questions, which have been immediately approved without the need for further discussion. The entire process, including the overall results of the vote, is reported in Figure S1.

The approved questions and their related approved critical outcomes are reported in Table 1.

The evidence review team identified the characteristics of relevant studies for each critical outcome, defining search strategy and study inclusion criteria, which are reported in Table S2. The search strategy used for all questions is “obesity AND surgery”, restricted to RCTs, with an expected start date of 1 June 2024.

A pre-specified analysis including trials performing MBS add-on to either structured lifestyle intervention (LSI), approved obesity management medications (OMM), or endoscopic surgery (at least 10 trials) will be performed, comparing those RCTs with others performed on MBS only in comparison with the standard of care. To compare these two latter subgroups of trials, we decided to use a “case-control” design. By matching with a 1:1 ratio, RCTs performed using MBS in add-on to other anti-obesity strategies with trials using MBS alone (i.e., MBS add-on to LSI vs. LSI or MBS add-on to an OMM versus SoC/Placebo or MBS add-on to endoscopic bariatric treatments versus SoC/Placebo). Matching parameters considered will be the same type of surgical intervention, mean BMI at trial entry date ± 2 Kg/m^2^, mean age at trial entry date ± 5 years, and duration of treatment ± 6 months. Trials used as “control study” will be chosen among trials included in a previous recent meta-analysis [19]. A study can serve, whenever needed, as the control for more than one “case study”.

## 4. Discussion

Obesity is a complex chronic disease requiring sustainable, multidimensional, and multimodal approaches. Strategies for adequate and effective treatment and management of this condition cannot be limited to structured lifestyle interventions and mostly require pharmacological, endoscopic, and surgical bariatric treatments [5,6,7,8,9]. These considerations are widely acknowledged and generally accepted by all professionals involved in obesity management; however, tailoring these strategies to the individual patient and deciding when employing a multimodal approach for different patient subpopulations affected by obesity is much more challenging. Candidates for bariatric and metabolic surgery often undergo multiple treatments for weight loss, but such treatments are often used as alternative therapies. Not to mention, by taking into consideration all RCT included in a recently published meta-analysis performed for the development of the Italian Guidelines for the surgical treatment of obesity, a structured lifestyle intervention was added to bariatric and metabolic surgery only in a small fraction of studies (12/61, 19.6%) [7]. Nevertheless, a larger proportion of studies regarding the effect of the endoscopic bariatric treatment (i.e., endoscopic weight loss interventions or endoscopic bariatric procedures) collected for the development of the Italian Guidelines for the endoscopic bariatric treatment of obesity adopted a structured intervention for the modification of lifestyle as add-on therapy (17/23, 74%; unpublished data) [30]. However, the underuse of nonpharmacological interventions in patients who are candidates for bariatric and metabolic surgery is a cause for concern and deserves vigorous advocacy for the selection of the appropriate individual therapeutic approach. Pharmacological treatments for obesity include several approved OMMs such as orlistat, lorcaserin, phentermine/topiramate, naltrexone/bupropion, liraglutide, semaglutide, and tirzepatide [10]. There are many studies and meta-analyses assessing the (short/medium-term) efficacy of pharmacological treatments in reducing body weight and some obesity-related medical conditions [31]. Few trials evaluate the effectiveness and efficacy of OMMs in patients with insufficient weight loss and weight regain after MBS, and even a smaller number of pilot studies attempt to explore the possibility of a combined surgical and pharmacological treatment in candidates for MBS [18,19,20]. Similar considerations can be made for endoscopic bariatric treatment adopted in patients undergoing MBS. The scarce literature on this strategy is either on “bridge treatments” in patients affected by severe obesity or suboptimal outcomes after other nonsurgical approaches [32,33]; only in a few studies has endoscopic bariatric treatment been regarded as a “multimodal” approach for the treatment of obesity (i.e., pre-planned endoscopic bariatric treatment add-on to subsequent MBS, irrespective of initial BMI) with conflicting results [21,34].

The IFSO-EC recognized the need for adopting nonsurgical strategies (i.e., lifestyle interventions, anti-obesity drugs, etc.) in patients requiring MBS and decided to develop a formal guideline aimed at making healthcare professionals aware of the possibility of a multimodal approach for the treatment of obesity and associated medical problems.

## Figures and Tables

**Table 1:** Delphi survey results and outcomes approval process.

N	PICO	Disagreement (Score 1–2)	Agreement (Score 3–5)	Outcome (Median)	Approval
	**A. Indication for Surgery**				
**1**	**In patients with BMI ≥ 30 kg/m^2^ and indication to MBS, is a pre- and/or post-treatment with structured lifestyle interventions preferable MBS alone, for the treatment of obesity?**	12.8%	87.2%	-	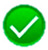
	*Outcomes* (efficacy)				
1.1	Body weight reduction (BMI; reduction in total body weight and excess body weight lost)			7.4	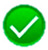
1.2	Improvement of glycometabolic control (glycosilated hemoglobin (HbA1c); fasting plasma glucose (FPG); and lipid and blood pressure profile)			7.5	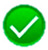
1.3	Comorbid conditions remission (diabetes, hypertension, dyslipidemia, OSAS, and arthropathy)			8.1	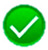
1.4	Reduction of all-cause mortality			7.6	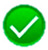
1.5	Improvement of quality of life			7.8	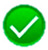
	*Outcomes* (safety)				
1.6	Perioperative surgical complications			8.2	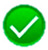
1.7	Serious adverse events (surgical and non-surgical)			8.3	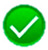
**2**	**In patients with BMI ≥ 30 kg/m^2^ and indication to MBS, is a pre- and/or post-treatment with approved obesity management medications preferable to MBS alone, for the treatment of obesity?**	16.7%	83.3%	-	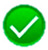
	*Outcomes* (efficacy)				
2.1	Body weight reduction (BMI; reduction in total body weight and excess body weight loss)			7.8	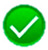
2.2	Improvement of glycometabolic control (glycosilated hemoglobin (HbA1c); fasting plasma glucose (FPG); and lipid and blood pressure profile)			8.1	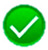
2.3	Comorbid conditions remission (diabetes, hypertension, dyslipidemia, OSAS, and arthropathy)			8.4	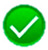
2.4	Reduction of all-cause mortality			8.2	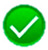
2.5	Improvement of quality of life			7.5	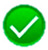
	*Outcomes* (safety)				
2.6	Perioperative surgical complications			7.9	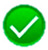
2.7	Serious adverse events (surgical and non-surgical)			8.3	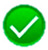
**3**	**In patients with BMI ≥ 30 kg/m^2^ and indication to MBS, is a pre-treatment with endoscopic bariatric interventions preferable to MBS alone, for the treatment of obesity?**	33.3%	66.7%	-	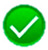
	*Outcomes* (efficacy)				
3.1	Body weight reduction (BMI; reduction in total body weight and excess body weight lost)			7.7	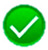
3.2	Improvement of glycometabolic control (glycosilated hemoglobin (HbA1c); fasting plasma glucose (FPG); and lipid and blood pressure profile)			7.5	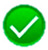
3.3	Comorbid conditions remission (diabetes, hypertension, dyslipidemia, OSAS, and arthropathy)			8.0	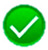
3.4	Reduction of all-cause mortality			7.9	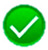
3.5	Improvement of quality of life			7.4	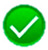
	*Outcomes* (safety)				
3.6	Perioperative surgical complications			8.0	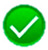
3.7	Serious adverse events (surgical and non-surgical)			8.3	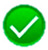

## Data Availability

The data presented in this study are available on request from the corresponding author.

## Supplementary Materials

The following supporting information can be downloaded at: https://www.mdpi.com/article/10.3390/jcm13175106/s1, Figure S1: overall results of the voting system with SurveyMonkey; Table S1: Characteristics and tasks of all panelists; Table S2: Approved (≥7 points) critical outcomes and main characteristics of eligible studies to be included in the upcoming meta-analyses.
