# Supplementary material for: Development of the International Federation for Surgery of Obesity and Metabolic Disorders-European Chapter (IFSO-EC) Grade-Based Guidelines on the Surgical Treatment of Obesity Using Multimodal Strategies: Design and Methodological Aspects
Source: J Clin Med. 2024 Aug 28;13(17):5106. doi: 10.3390/jcm13175106 (PMC11396011; doi:10.3390/jcm13175106)
Supplement: Supplementary file 1 [file jcm-13-05106-s001.zip › Figure S1.pdf]

Q1 What’s your name?

Answered: 23 Skipped: 1

#	RESPONSES	DATE
1	Di Lorenzo	6/17/2024 9:19 PM
2	Catalin Copaescu	6/16/2024 10:51 PM
3	Marloes Emous	6/16/2024 9:24 PM
4	RAMON VILALLONGA	6/16/2024 8:15 PM
5	Christine	6/16/2024 7:50 PM
6	Rui Ribeiro	6/13/2024 8:03 PM
7	Nasser Sakran	6/13/2024 6:15 AM
8	Gerhard Prager	6/13/2024 1:19 AM
9	Halit Eren Taskin	6/12/2024 7:28 AM
10	juan pujol Rafols	6/11/2024 8:25 PM
11	Simon Nienhuijs	6/8/2024 7:51 PM
12	Chetan Parmar	6/7/2024 11:00 PM
13	Matteo Monami	6/6/2024 12:56 PM
14	Martin Fried	6/6/2024 10:43 AM
15	Amanda Belluzzi	6/6/2024 8:34 AM
16	Marco Bueter	6/6/2024 12:17 AM
17	Daniel Moritz Felsenreich	6/5/2024 11:52 AM
18	Francesco Maria Carrano	6/5/2024 12:01 AM
19	Elena Ruiz-Úcar	6/4/2024 10:56 PM
20	Erik Stenberg	6/4/2024 9:25 PM
21	Sonja Chiappetta	6/4/2024 7:16 PM
22	Paulina Salminen	6/4/2024 7:14 PM
23	Maurizio De Luca	6/4/2024 6:27 PM

Q2 PICO 1 In patients with BMI >=30 kg/m2 and indication to bariatric and metabolic surgery, is a pre- and/or post-treatment with structured lifestyle interventions preferable to bariatric and metabolic surgical alone, for the treatment of obesity?1= unuseful; 5= very important

Answered: 24 Skipped: 0

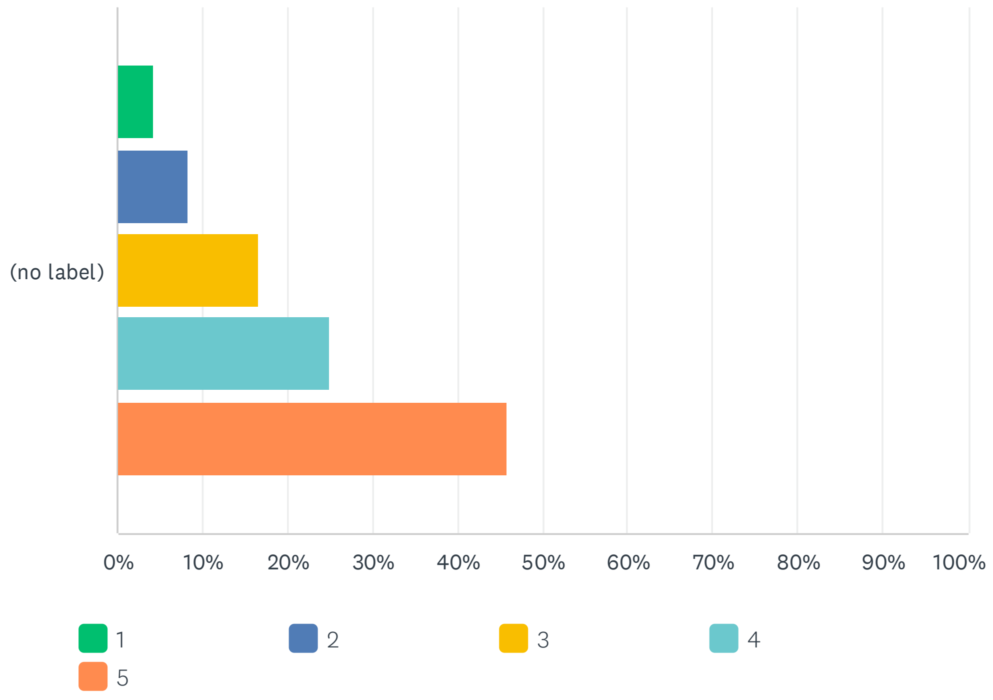

	1	2	3	4	5	TOTAL	WEIGHTED AVERAGE
(no label)	4.17%<br>1	8.33%<br>2	16.67%<br>4	25.00%<br>6	45.83%<br>11	24	4.00

Q3 PICO 1 (Outcome 1) Improvement of glycometabolic control (glycosilated heamoglobin (HbA1c); fasting plasma glucose (FPG); lipid profile; systolic blood pressure (SBP), diastolic blood pressure (DBP))1-6= not critical; 7-9= critical

Answered: 24 Skipped: 0

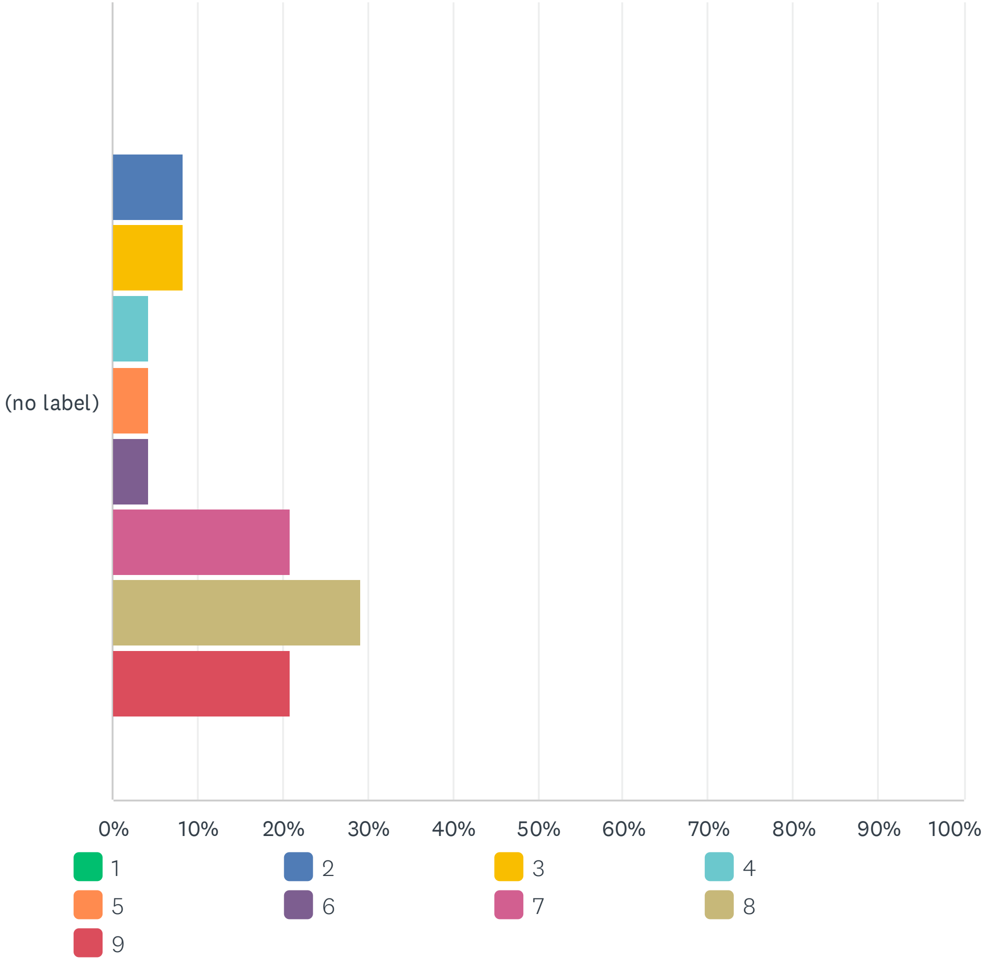

	1	2	3	4	5	6	7	8	9	TOTAL	WEIGHTED AVERAGE
(no label)	0.00%<br>0	8.33%<br>2	8.33%<br>2	4.17%<br>1	4.17%<br>1	4.17%<br>1	20.83%<br>5	29.17%<br>7	20.83%<br>5	24	7.46

Q4 PICO 1 (Outcome 2) Body weight reduction (BMI; TWL, EBWL)1-6= not critical; 7-9= critical

Answered: 24 Skipped: 0

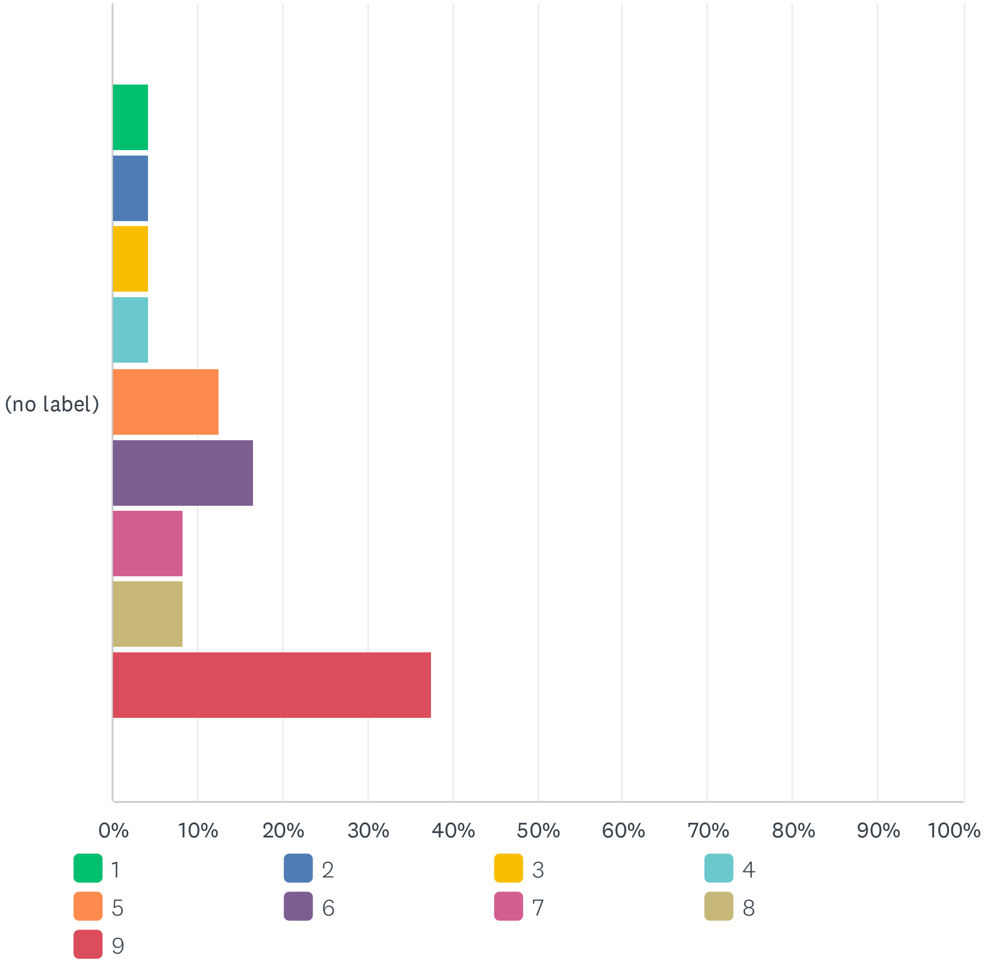

	1	2	3	4	5	6	7	8	9	TOTAL	WEIGHTED AVERAGE
(no label)	4.17%<br>1	4.17%<br>1	4.17%<br>1	4.17%<br>1	12.50%<br>3	16.67%<br>4	8.33%<br>2	8.33%<br>2	37.50%<br>9	24	7.38

Q5 PICO 1 (Outcome 3) Reduction of all-cause mortality1-6= not critical; 7-9= critical

Answered: 24 Skipped: 0

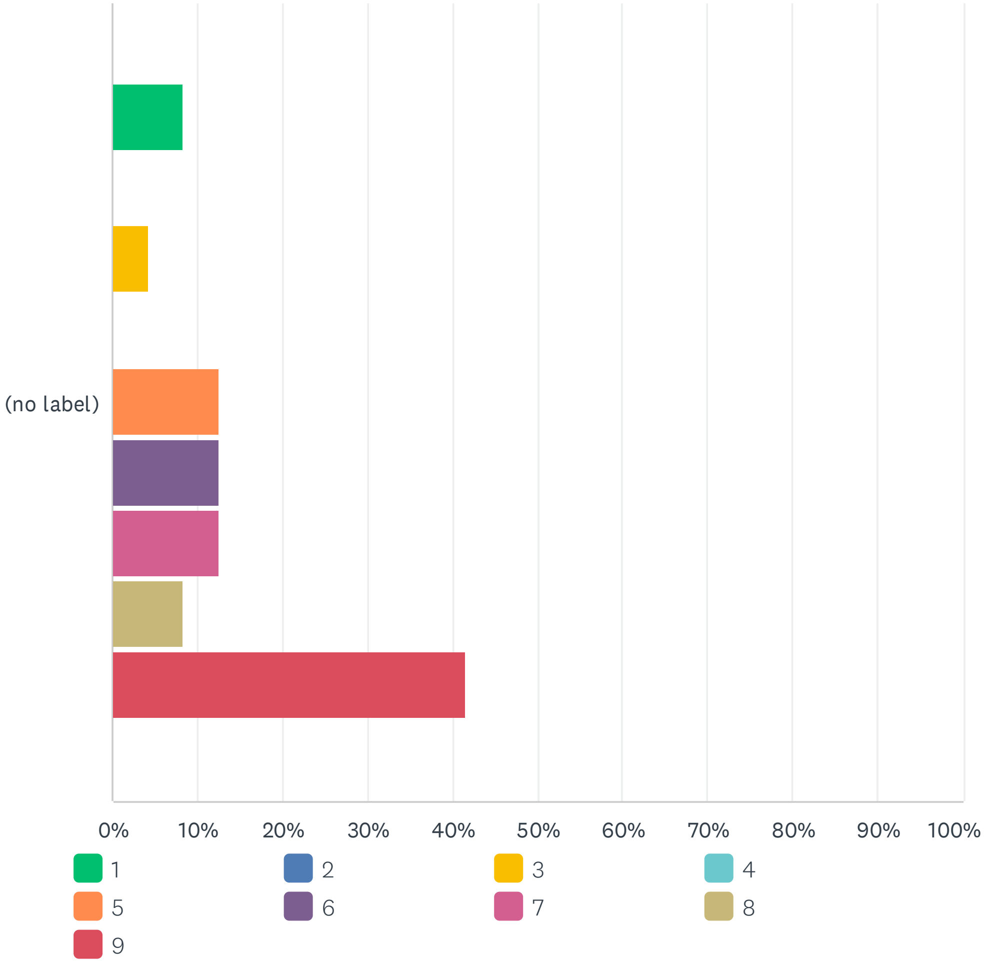

	1	2	3	4	5	6	7	8	9	TOTAL	WEIGHTED AVERAGE
(no label)	8.33% 2	0.00% 0	4.17% 1	0.00% 0	12.50% 3	12.50% 3	12.50% 3	8.33% 2	41.67% 10	24	7.63

Q6 PICO 1 (Outcome 4) Improvement of quality of life1-6= not critical; 7-9= critical

Answered: 24 Skipped: 0

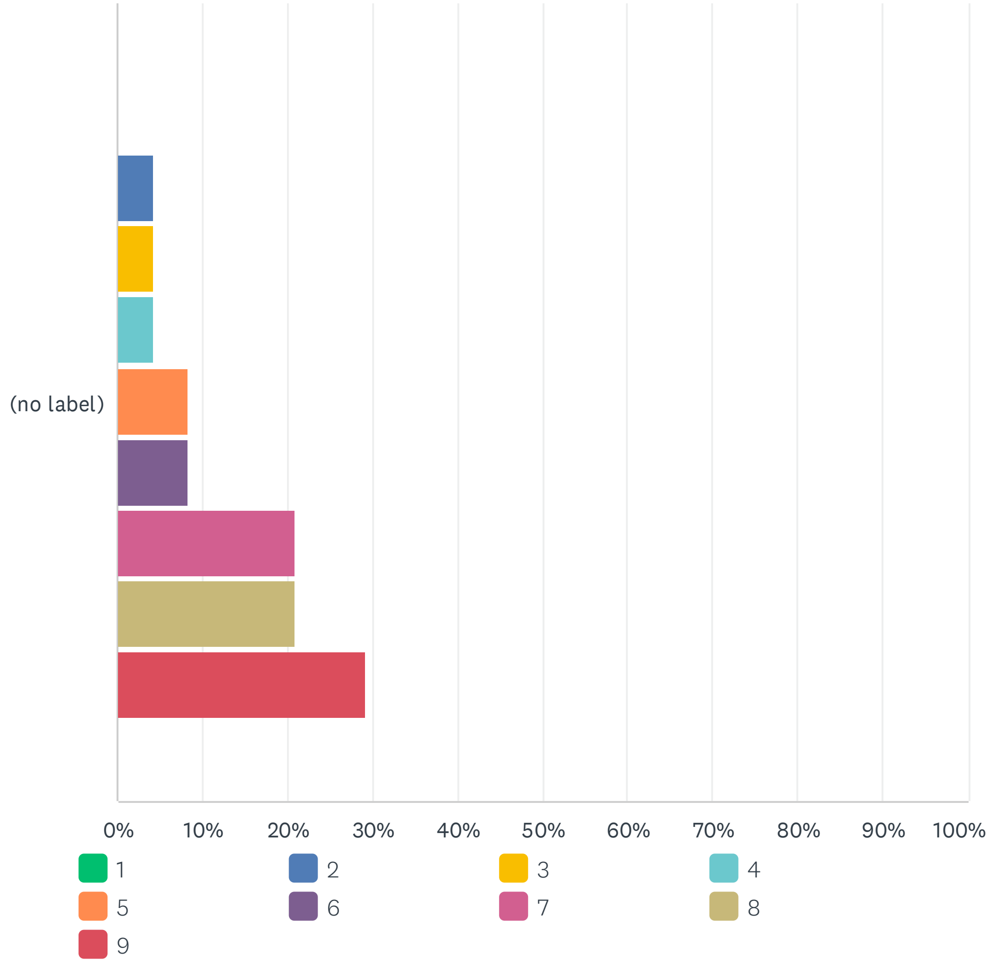

	1	2	3	4	5	6	7	8	9	TOTAL	WEIGHTED AVERAGE
(no label)	0.00% 0	4.17% 1	4.17% 1	4.17% 1	8.33% 2	8.33% 2	20.83% 5	20.83% 5	29.17% 7	24	7.83

Q7 PICO 1 (Outcome 5) Comorbid conditions remission (Diabetes hypertension, dyslipidemia, OSAS)1-6= not critical; 7-9= critical

Answered: 24 Skipped: 0

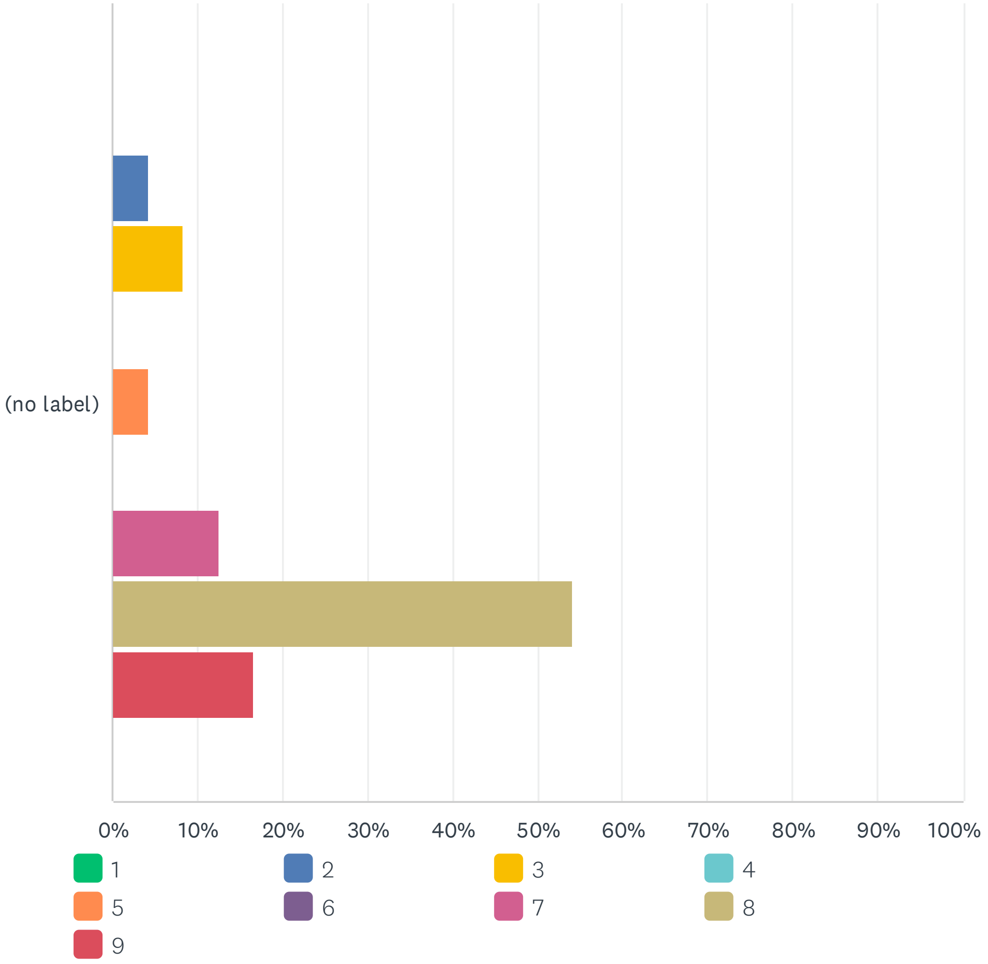

	1	2	3	4	5	6	7	8	9	TOTAL	WEIGHTED AVERAGE
(no label)	0.00%<br>0	4.17%<br>1	8.33%<br>2	0.00%<br>0	4.17%<br>1	0.00%<br>0	12.50%<br>3	54.17%<br>13	16.67%<br>4	24	8.08

Q8 PICO 1 (Outcome 7; safety) Perioperative surgical complications 1-6= not critical; 7-9= critical

Answered: 24 Skipped: 0

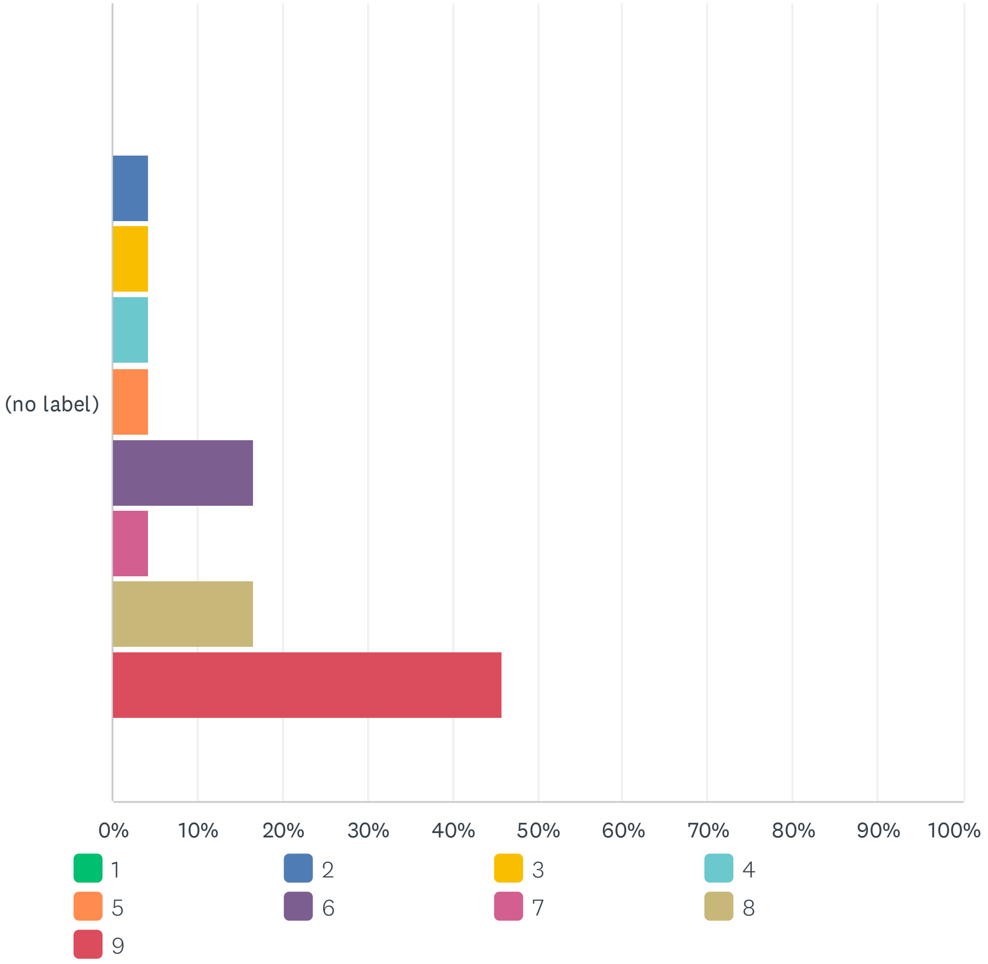

	1	2	3	4	5	6	7	8	9	TOTAL	WEIGHTED AVERAGE
(no label)	0.00%<br>0	4.17%<br>1	4.17%<br>1	4.17%<br>1	4.17%<br>1	16.67%<br>4	4.17%<br>1	16.67%<br>4	45.83%<br>11	24	8.17

Q9 PICO 1 (Outcome 8; safety) Overall SAE. 1-6= not critical; 7-9= critical

Answered: 24 Skipped: 0

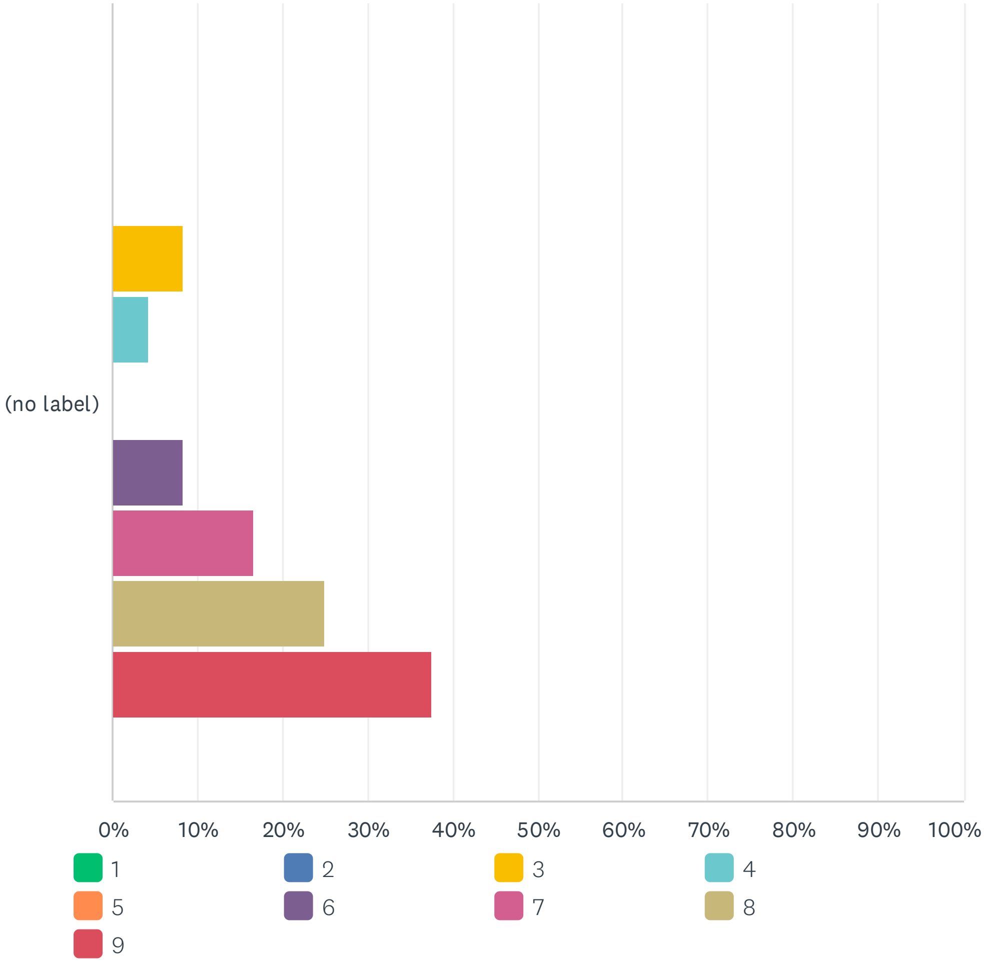

	1	2	3	4	5	6	7	8	9	TOTAL	WEIGHTED AVERAGE
(no label)	0.00%<br>0	0.00%<br>0	8.33%<br>2	4.17%<br>1	0.00%<br>0	8.33%<br>2	16.67%<br>4	25.00%<br>6	37.50%<br>9	24	8.33

Q10 PICO 2 In patients with BMI >=30 kg/m2 and indication to bariatric and metabolic surgery, is a pre- and/or post-treatment with approved anti-obesity drugs preferable to bariatric and metabolic surgical alone, for the treatment of obesity?1= unuseful; 5= very important

Answered: 24 Skipped: 0

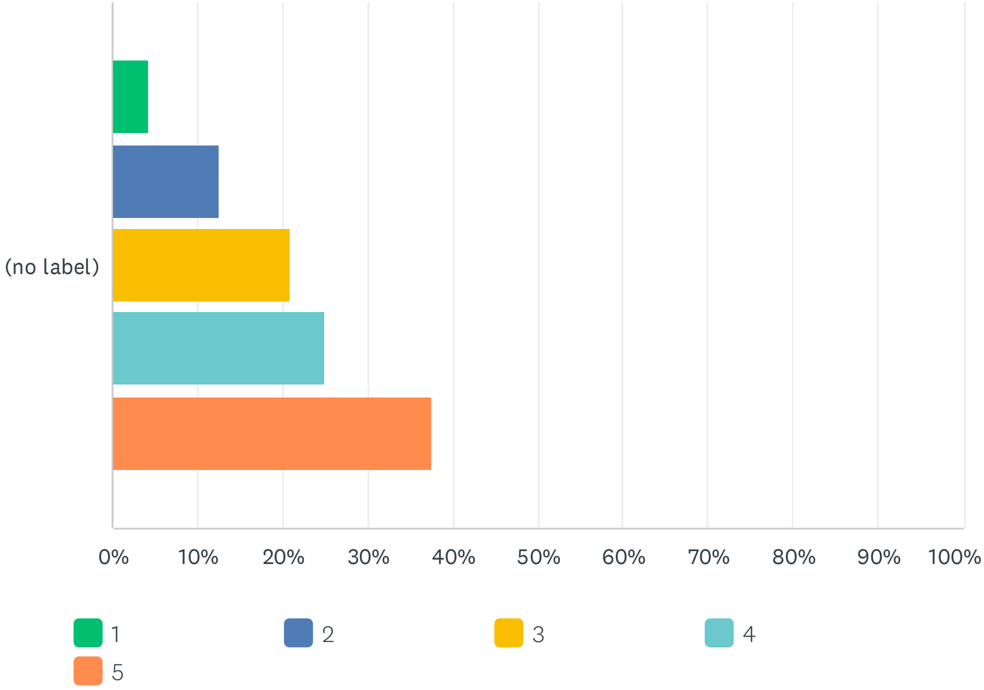

	1	2	3	4	5	TOTAL	WEIGHTED AVERAGE
(no label)	4.17% 1	12.50% 3	20.83% 5	25.00% 6	37.50% 9	24	3.79

Q11 PICO 2 (Outcome 1) Improvement of glycometabolic control (glycosilated heamoglobin (HbA1c); fasting plasma glucose (FPG); lipid profile; systolic blood pressure (SBP), diastolic blood pressure (DBP))1-6= not critical; 7-9= critical

Answered: 24 Skipped: 0

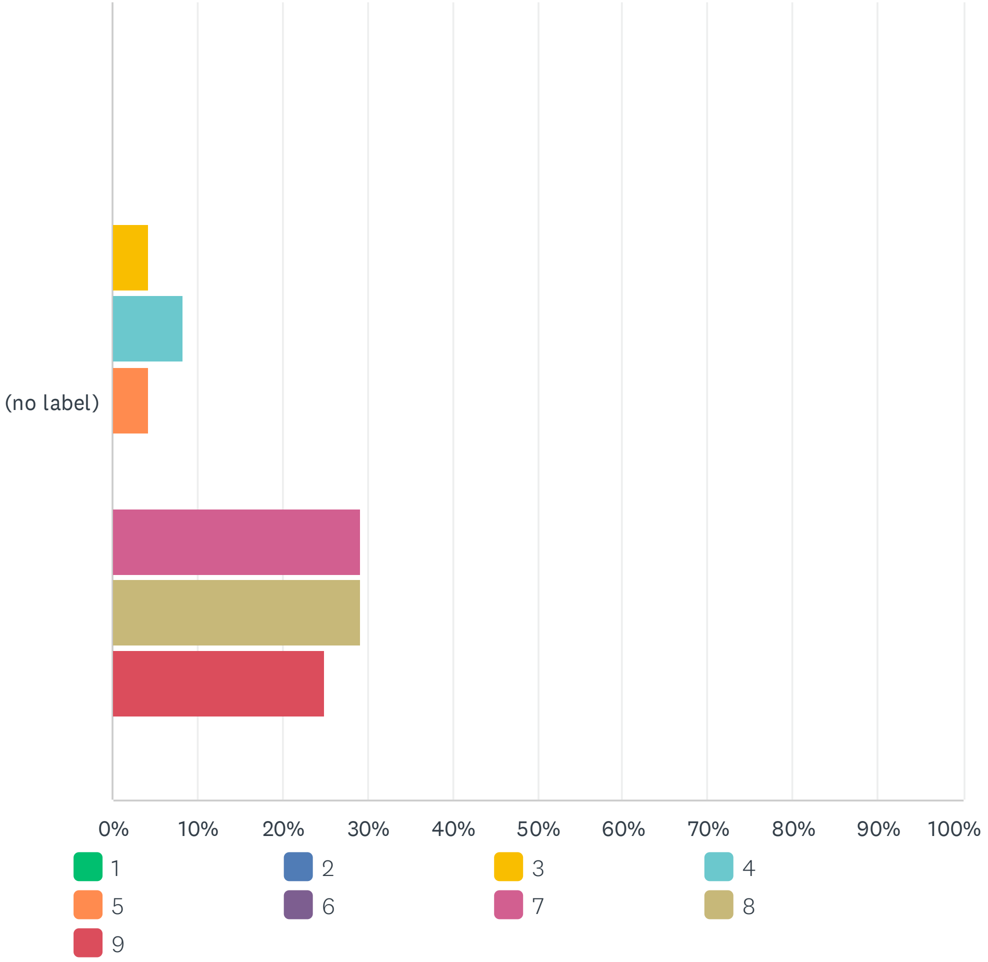

	1	2	3	4	5	6	7	8	9	TOTAL	WEIGHTED AVERAGE
(no label)	0.00% 0	0.00% 0	4.17% 1	8.33% 2	4.17% 1	0.00% 0	29.17% 7	29.17% 7	25.00% 6	24	8.13

Q12 PICO 2 (Outcome 2) Body weight reduction (BMI; TWL, EBWL)1-6= not critical; 7-9= critical

Answered: 24 Skipped: 0

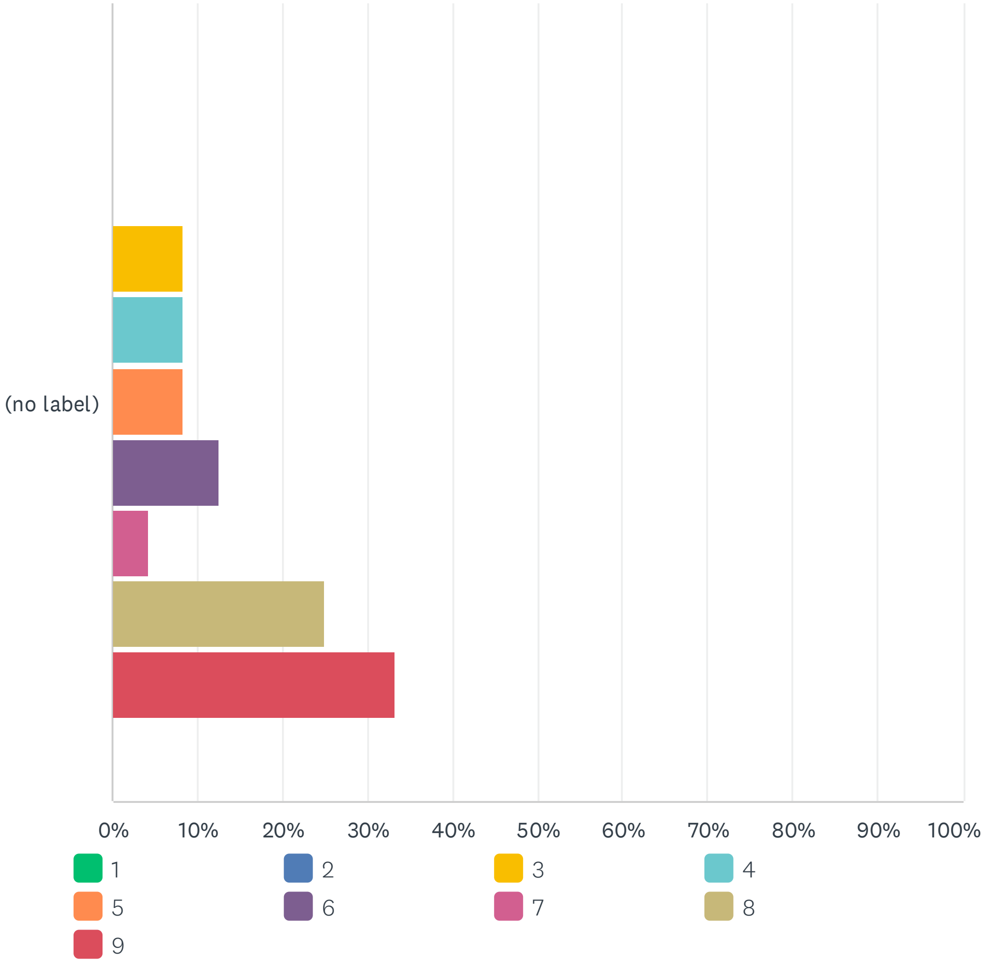

	1	2	3	4	5	6	7	8	9	TOTAL	WEIGHTED AVERAGE
(no label)	0.00% 0	0.00% 0	8.33% 2	8.33% 2	8.33% 2	12.50% 3	4.17% 1	25.00% 6	33.33% 8	24	7.79

Q13 PICO 2 (Outcome 3) Reduction of all-cause mortality1-6= not critical; 7-9= critical

Answered: 24 Skipped: 0

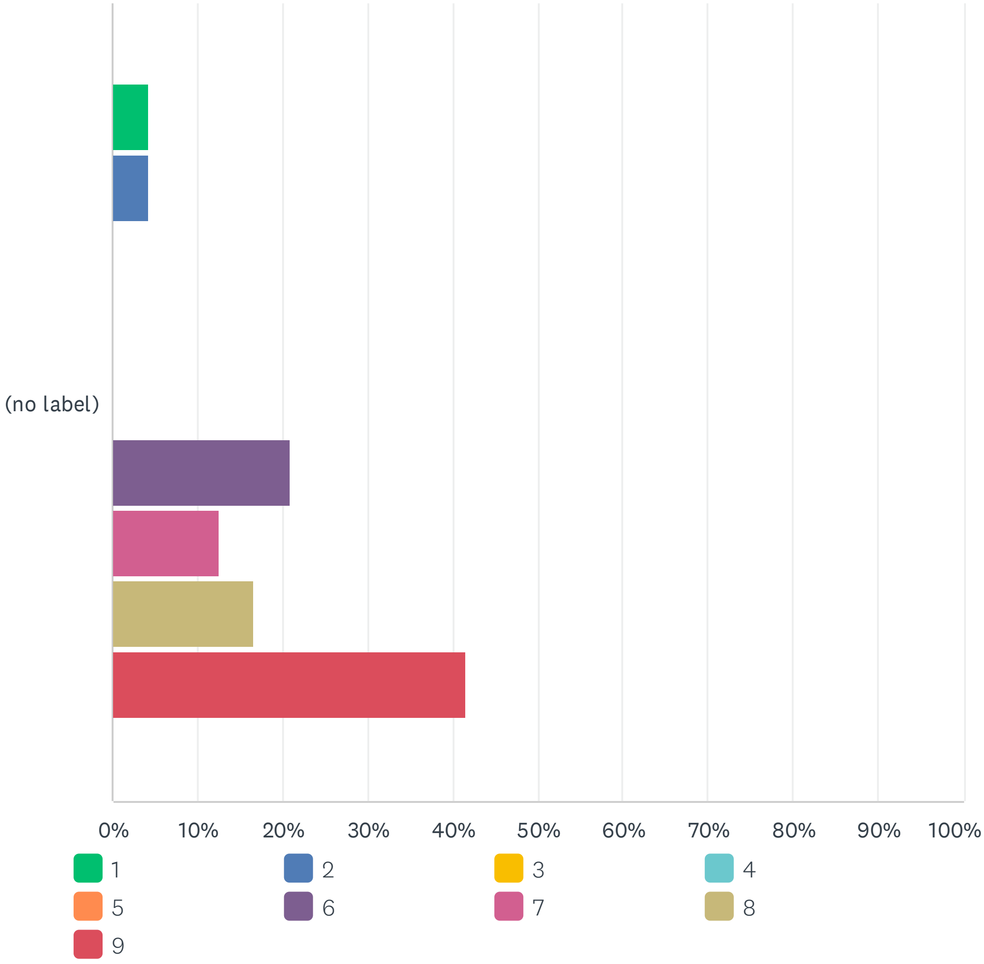

	1	2	3	4	5	6	7	8	9	TOTAL	WEIGHTED AVERAGE
(no label)	4.17% 1	4.17% 1	0.00% 0	0.00% 0	0.00% 0	20.83% 5	12.50% 3	16.67% 4	41.67% 10	24	8.25

Q14 PICO 2 (Outcome 4) Improvement of quality of life1-6= not critical; 7-9= critical

Answered: 24 Skipped: 0

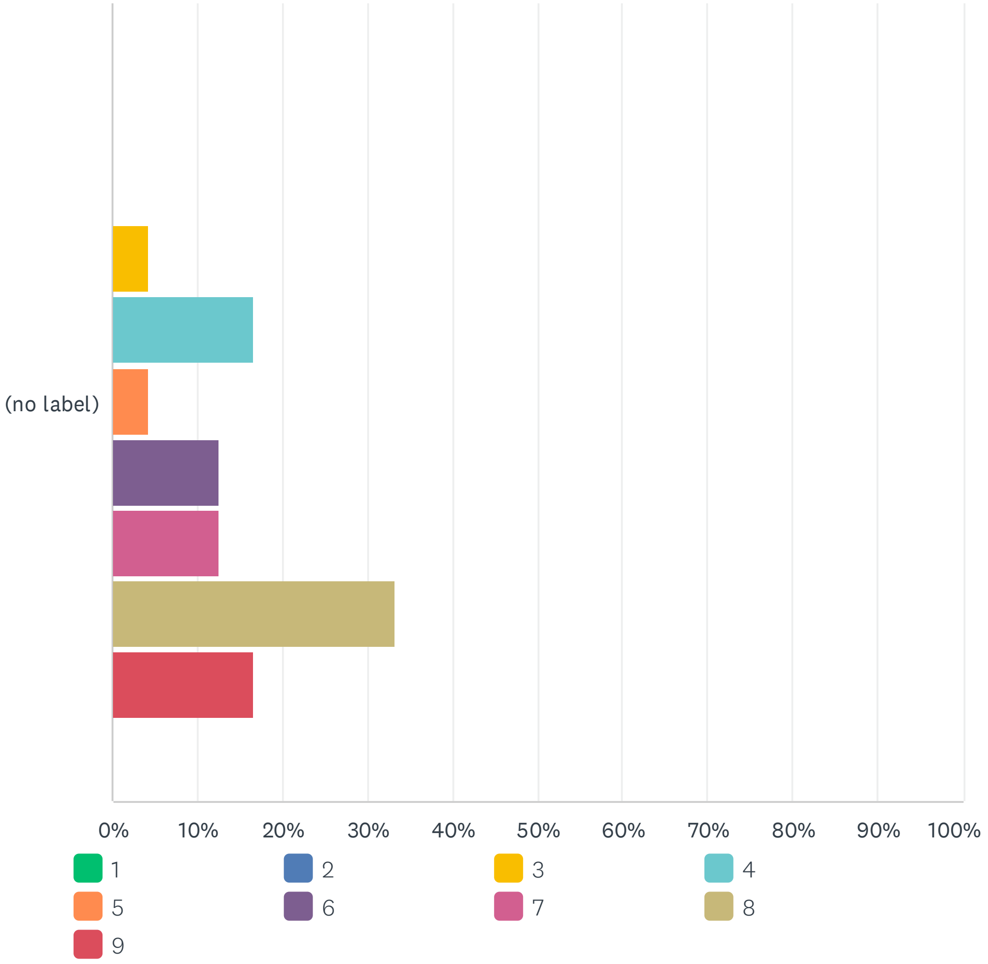

	1	2	3	4	5	6	7	8	9	TOTAL	WEIGHTED AVERAGE
(no label)	0.00% 0	0.00% 0	4.17% 1	16.67% 4	4.17% 1	12.50% 3	12.50% 3	33.33% 8	16.67% 4	24	7.54

Q15 PICO 2 (Outcome 5) Comorbid conditions remission (Diabetes hypertension, dyslipidemia, OSAS, arthropathy)1-6= not critical; 7-9= critical

Answered: 24 Skipped: 0

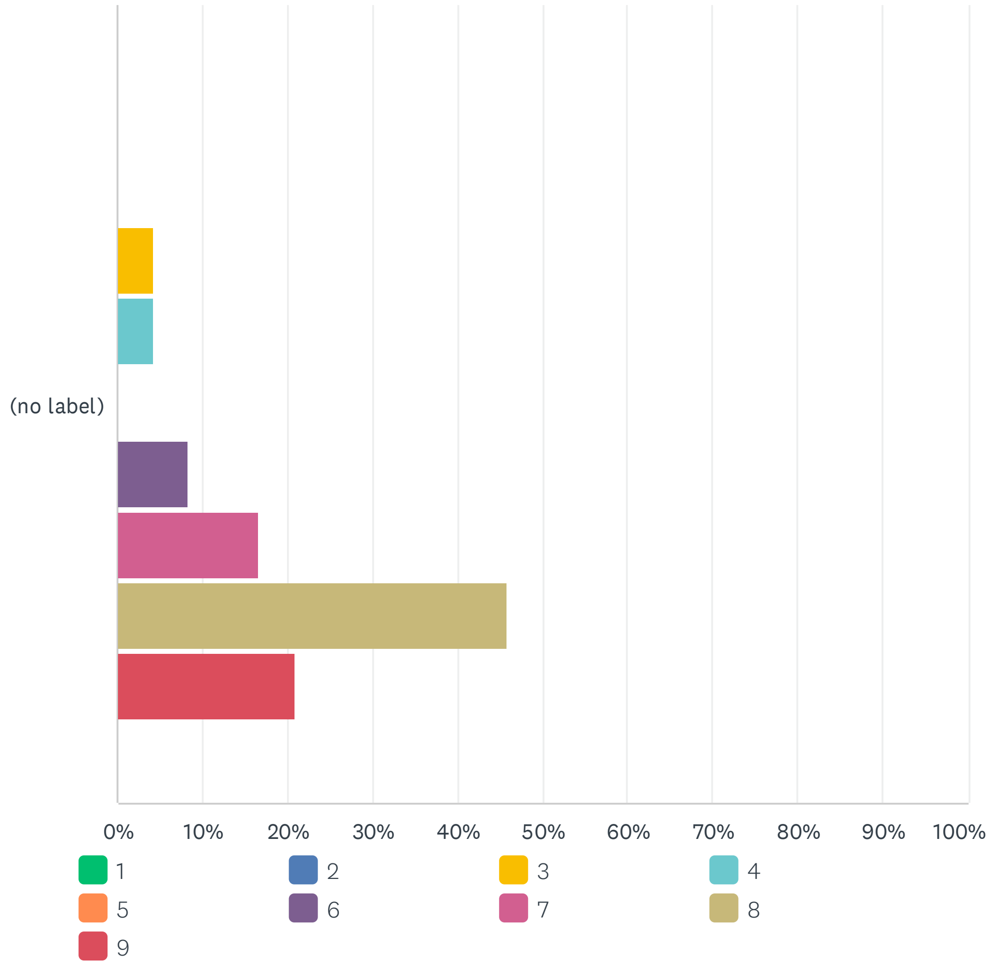

	1	2	3	4	5	6	7	8	9	TOTAL	WEIGHTED AVERAGE
(no label)	0.00%<br>0	0.00%<br>0	4.17%<br>1	4.17%<br>1	0.00%<br>0	8.33%<br>2	16.67%<br>4	45.83%<br>11	20.83%<br>5	24	8.42

Q16 PICO 2 (Outcome 7; safety) Perioperative surgical complications 1-6= not critical; 7-9= critical

Answered: 24 Skipped: 0

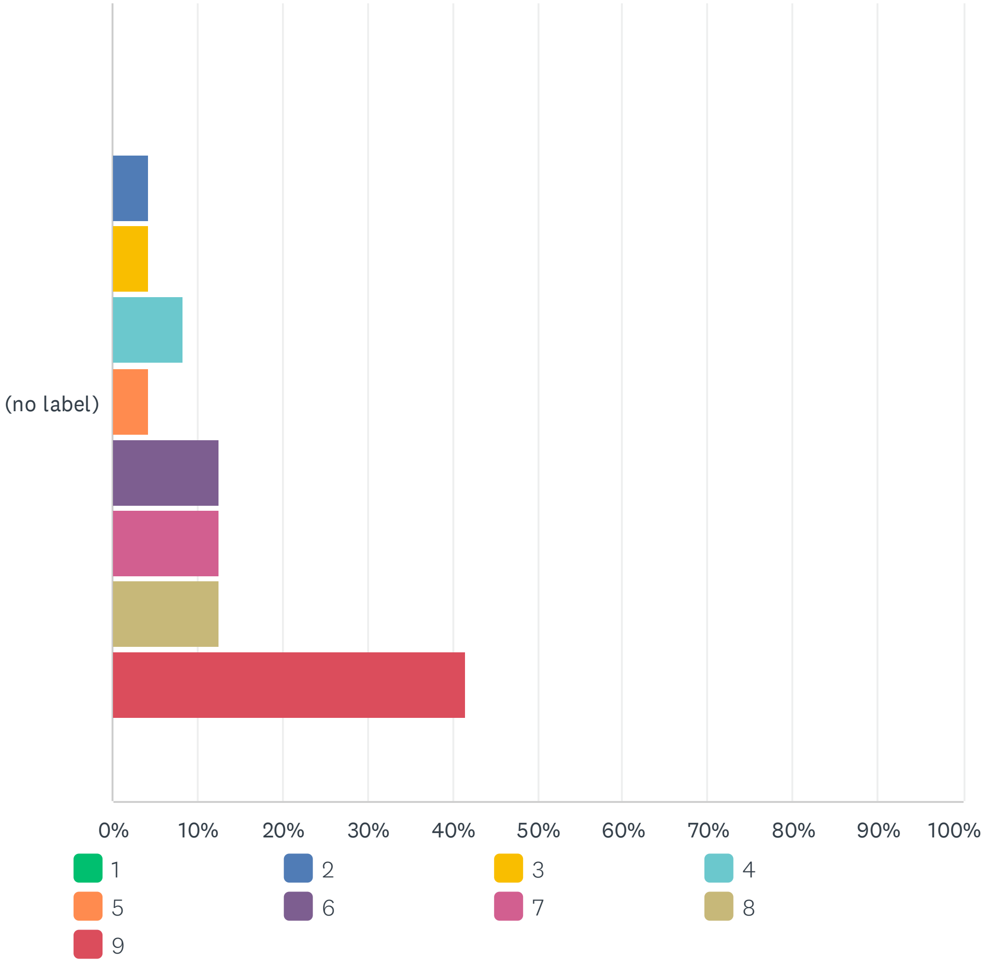

	1	2	3	4	5	6	7	8	9	TOTAL	WEIGHTED AVERAGE
(no label)	0.00% 0	4.17% 1	4.17% 1	8.33% 2	4.17% 1	12.50% 3	12.50% 3	12.50% 3	41.67% 10	24	7.92

Q17 PICO 2 (Outcome 8; safety) Overall SAE. 1-6= not critical; 7-9= critical

Answered: 23 Skipped: 1

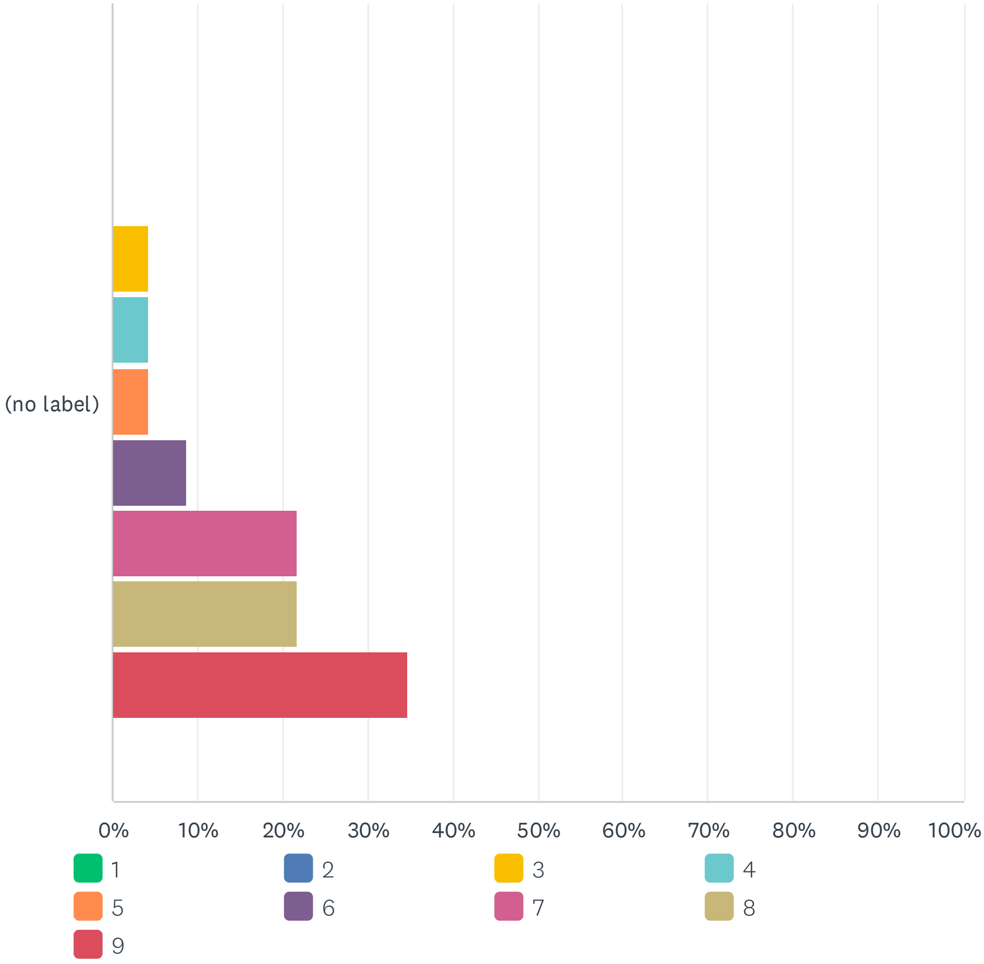

	1	2	3	4	5	6	7	8	9	TOTAL	WEIGHTED AVERAGE
(no label)	0.00% 0	0.00% 0	4.35% 1	4.35% 1	4.35% 1	8.70% 2	21.74% 5	21.74% 5	34.78% 8	23	8.30

Q18 PICO 3 In patients with BMI >=30 kg/m2 and indication to bariatric and metabolic surgery, is a pre- and/or post-treatment with endobariatric surgery preferable to bariatric and metabolic surgical alone, for the treatment of obesity?1= unuseful; 5= very important

Answered: 24 Skipped: 0

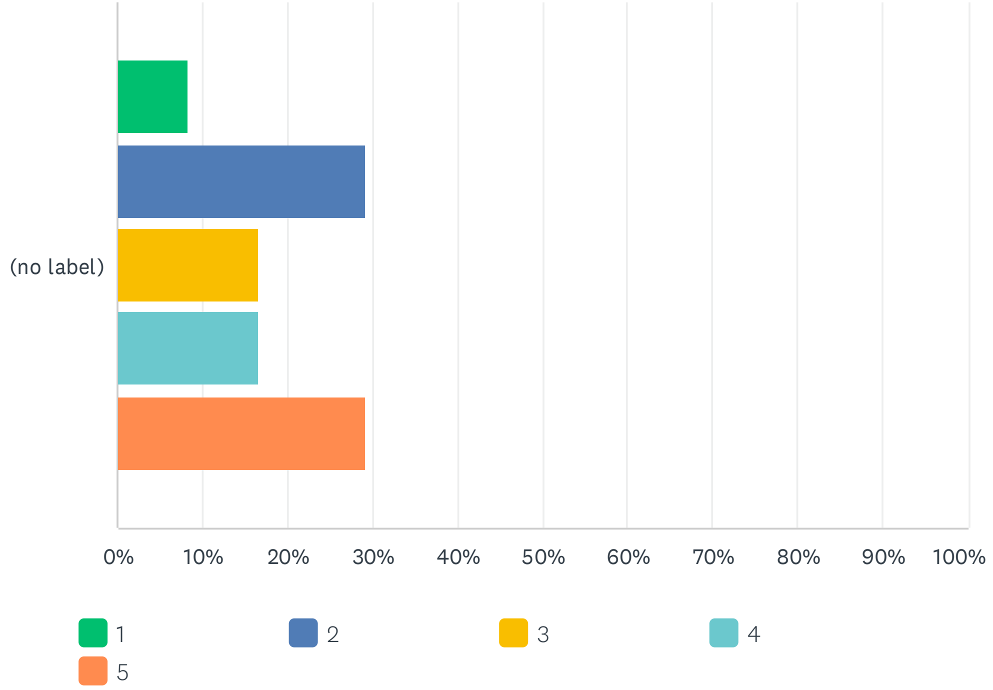

	1	2	3	4	5	TOTAL	WEIGHTED AVERAGE
(no label)	8.33%<br>2	29.17%<br>7	16.67%<br>4	16.67%<br>4	29.17%<br>7	24	3.29

Q19 PICO 3 (Outcome 1) Improvement of glycometabolic control (glycosilated heamoglobin (HbA1c); fasting plasma glucose (FPG); lipid profile; systolic blood pressure (SBP), diastolic blood pressure (DBP))1-6= not critical; 7-9= critical

Answered: 24 Skipped: 0

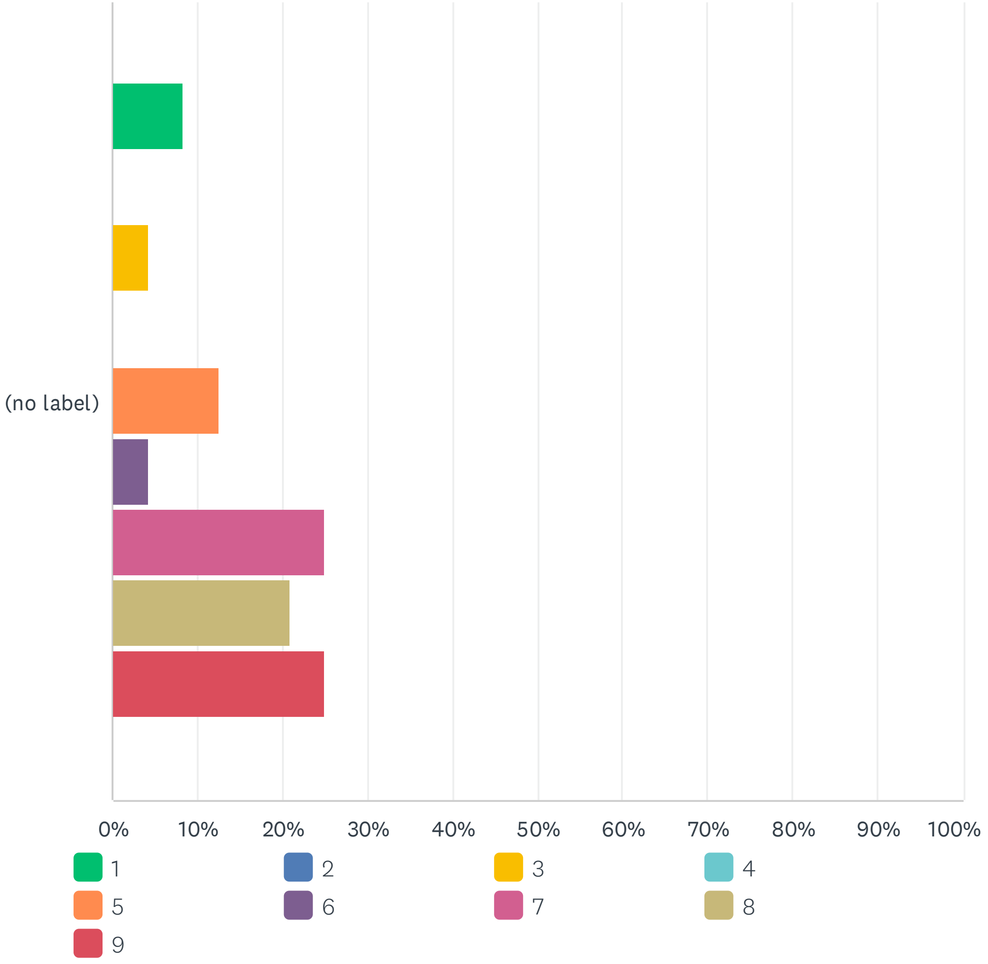

	1	2	3	4	5	6	7	8	9	TOTAL	WEIGHTED AVERAGE
(no label)	8.33% 2	0.00% 0	4.17% 1	0.00% 0	12.50% 3	4.17% 1	25.00% 6	20.83% 5	25.00% 6	24	7.50

Q20 PICO 3 (Outcome 2) Body weight reduction (BMI; TWL, EBWL)1-6= not critical; 7-9= critical

Answered: 24 Skipped: 0

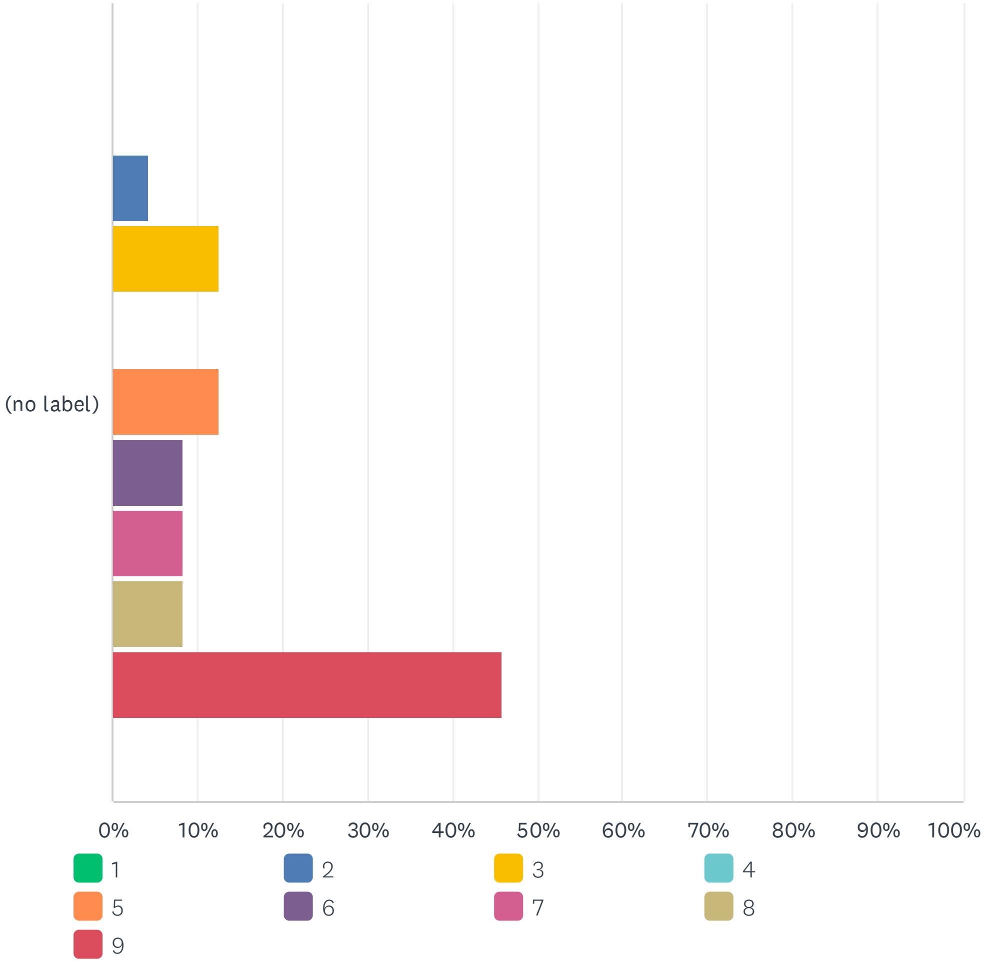

	1	2	3	4	5	6	7	8	9	TOTAL	WEIGHTED AVERAGE
(no label)	0.00%<br>0	4.17%<br>1	12.50%<br>3	0.00%<br>0	12.50%<br>3	8.33%<br>2	8.33%<br>2	8.33%<br>2	45.83%<br>11	24	7.67

Q21 PICO 3 (Outcome 3) Reduction of all-cause mortality1-6= not critical; 7-9= critical

Answered: 24 Skipped: 0

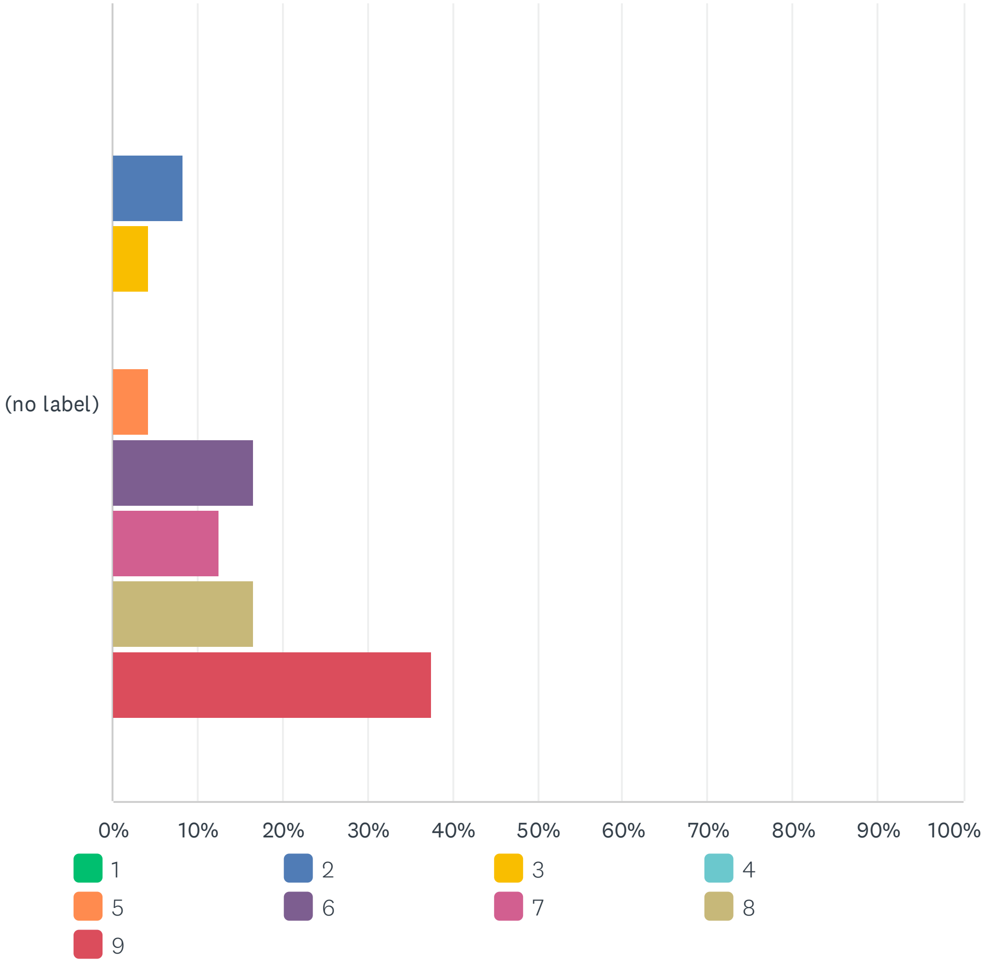

	1	2	3	4	5	6	7	8	9	TOTAL	WEIGHTED AVERAGE
(no label)	0.00%<br>0	8.33%<br>2	4.17%<br>1	0.00%<br>0	4.17%<br>1	16.67%<br>4	12.50%<br>3	16.67%<br>4	37.50%<br>9	24	7.92

Q22 PICO 3 (Outcome 4) Improvement of quality of life1-6= not critical; 7-9= critical

Answered: 24 Skipped: 0

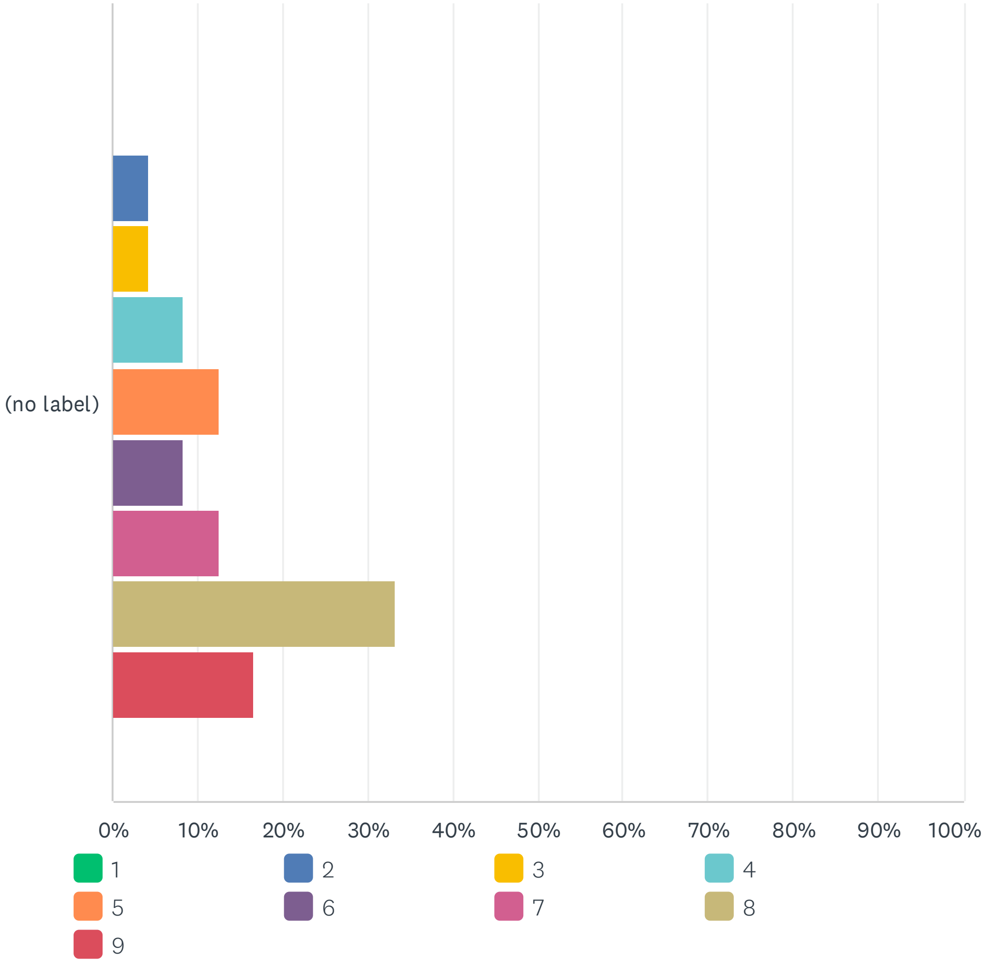

	1	2	3	4	5	6	7	8	9	TOTAL	WEIGHTED AVERAGE
(no label)	0.00% 0	4.17% 1	4.17% 1	8.33% 2	12.50% 3	8.33% 2	12.50% 3	33.33% 8	16.67% 4	24	7.42

Q23 PICO 3 (Outcome 5) Comorbid conditions remission (Diabetes hypertension, dyslipidemia, OSAS, arthropathy)1-6= not critical; 7-9= critical

Answered: 24 Skipped: 0

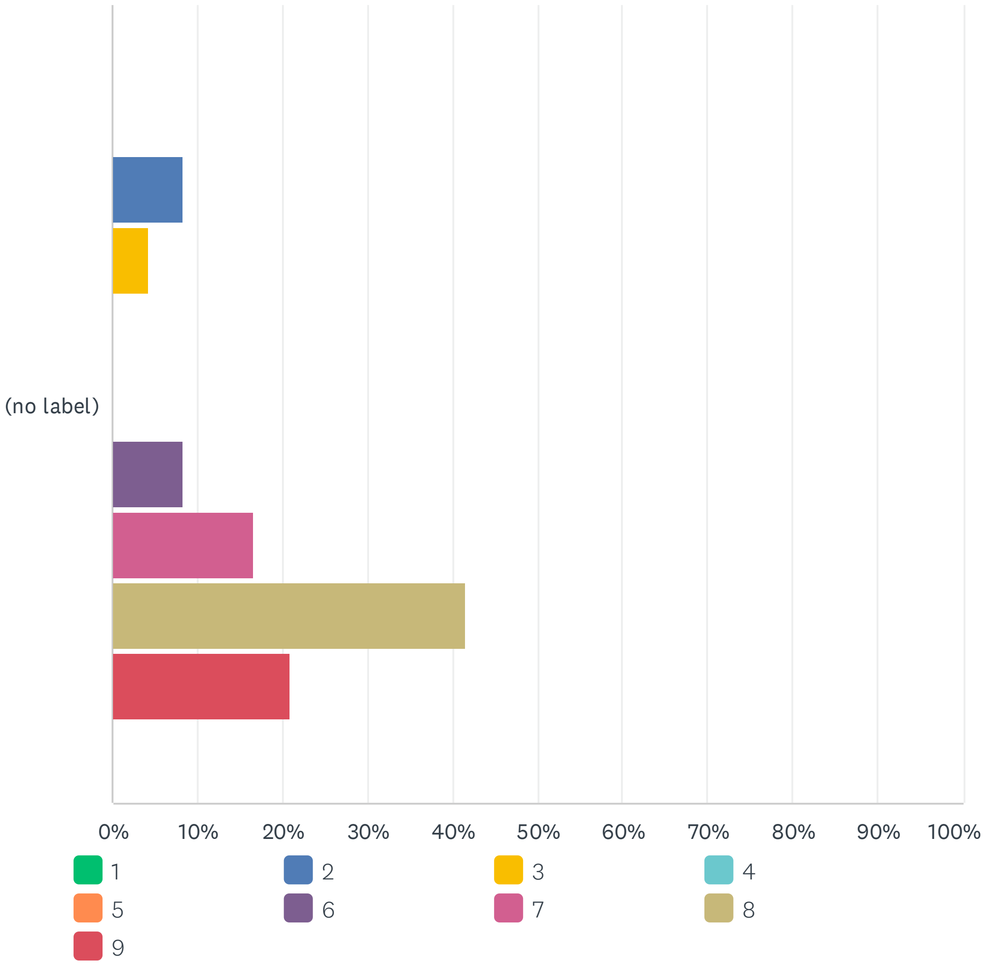

	1	2	3	4	5	6	7	8	9	TOTAL	WEIGHTED AVERAGE
(no label)	0.00%<br>0	8.33%<br>2	4.17%<br>1	0.00%<br>0	0.00%<br>0	8.33%<br>2	16.67%<br>4	41.67%<br>10	20.83%<br>5	24	8.04

Q24 PICO 3 (Outcome 7; safety) Perioperative surgical complications 1-6= not critical; 7-9= critical

Answered: 24 Skipped: 0

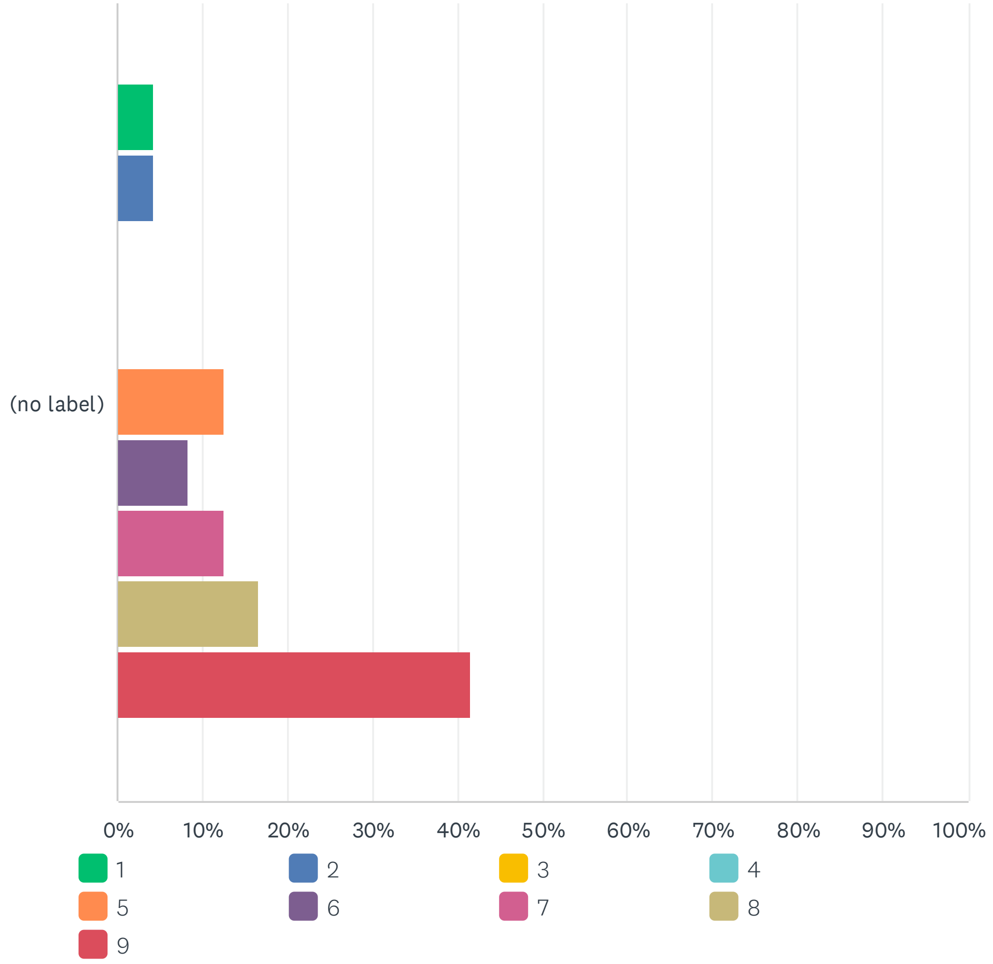

	1	2	3	4	5	6	7	8	9	TOTAL	WEIGHTED AVERAGE
(no label)	4.17% 1	4.17% 1	0.00% 0	0.00% 0	12.50% 3	8.33% 2	12.50% 3	16.67% 4	41.67% 10	24	8.00

Q25 PICO 3 (Outcome 8; safety) Overall SAE. 1-6= not critical; 7-9= critical

Answered: 24 Skipped: 0

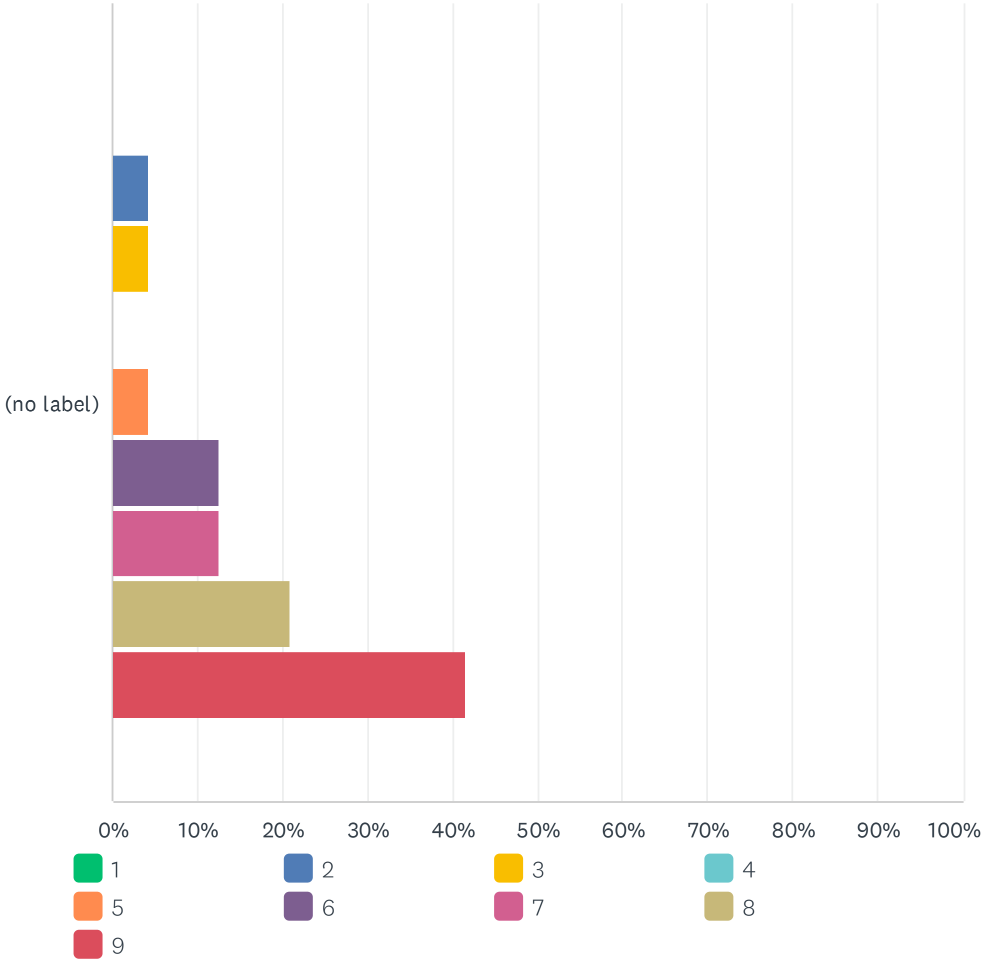

	1	2	3	4	5	6	7	8	9	TOTAL	WEIGHTED AVERAGE
(no label)	0.00% 0	4.17% 1	4.17% 1	0.00% 0	4.17% 1	12.50% 3	12.50% 3	20.83% 5	41.67% 10	24	8.33
