# Supplementary material for: Development of the International Federation for Surgery of Obesity and Metabolic Disorders-European Chapter (IFSO-EC) Grade-Based Guidelines on the Surgical Treatment of Obesity Using Multimodal Strategies: Design and Methodological Aspects
Source: J Clin Med. 2024 Aug 28;13(17):5106. doi: 10.3390/jcm13175106 (PMC11396011; doi:10.3390/jcm13175106)
Supplement: Supplementary file 1 [file jcm-13-05106-s001.zip › jcm-3167513-supplementary.pdf]

SUPPLEMENTARY MATERIALS:

Table S1 – Characteristics and tasks of all panelists

N	Name	Profession	Age	Gender	Role
1	Maurizio de Luca	Surgeon	57	M	Coordinator/Panelist
2	Paulina Salminen	Surgeon	53	F	Panelist
3	Marco Bueter	Surgeon	47	M	Panelist
4	Sonja Chiappetta	Surgeon	40	F	Panelist
5	Francesco Maria Carrano	Surgeon	36	M	Panelist
6	Erik Stenberg	Surgeon	42	M	Panelist
7	Daniel Moritz Felsenreich	Surgeon	40	M	Panelist
8	Amanda Belluzzi	Surgeon	31	F	Panelist
9	Martin Fried	Surgeon	65	M	Panelist
10	Elena Ruiz-Ucar	Surgeon	43	F	Panelist
11	Matteo Monami	Diabetologist	50	M	Methodologist/Panelist
12	Juan Pujol-Rafols	Surgeon	55	M	Panelist
13	Nasser Sakran	Surgeon	56	M	Panelist
14	Christine Stier	Surgeon	60	F	Panelist
15	Eren Halit Taskin	Surgeon	40	F	Panelist
16	Nicola Di Lorenzo	Surgeon	65	M	Panelist
17	Simon Nienhuijs	Surgeon		F	Panelist
18	Ramón Vilallonga Puy	Surgeon	48	M	Panelist

N	Name	Profession	Age	Gender	Role
19	Marloes Emous	Surgeon		F	Panelist
20	Gerhard Prager	Surgeon	56	M	Panelist
21	Jacques Himpens	Surgeon	71	M	Panelist
22	Antonio Iannelli	Surgeon	58	M	Panelist
23	Chetan Parmar	Surgeon	57	M	Panelist
24	Catalin Copaescu	Surgeon	61	M	Panelist
25	Iris Zani	Patient Representative	57	F	Panelist
26	Benedetta Ragghianti	Endocrinologist	45	F	Evidence reviewer
28	Daniele Scoccimarro	Endocrinologist	34	M	Evidence reviewer
29	Giulia Bandini	Internal Medicine	37	F	Evidence reviewer

Table S2 – Approved (≥ 7 points) critical outcomes and main characteristics of eligible studies to be included in the upcoming meta-analyses.

N	Outcome*	Type of study	Main inclusion criteria
1.1, 2.1, 3.1	Body weight reduction	RCT	Patients aged 18+ years, with obesity; studies performed on patients with BMI≥ 30 Kg/m^2 and duration≥ 16 weeks. BMI, percentage of weight loss and excess weight loss, and total body weight loss at endpoint.
1.2, 2.2, 3.2	Improvement of glycometabolic control	RCT	Patients aged 18+ years, with obesity; studies performed on patients with BMI≥ 30 Kg/m^2 and duration≥ 16 weeks. HbA1c; FPG, Improvement of glycometabolic control (glycosilated hemoglobin (HbA1c); fasting plasma glucose (FPG), and lipid and blood pressure profile) at endpoint.
1.3, 2.3, 3.3	Obesity-related medical condition remission	RCT	Patients aged ≥18 years, with obesity and at least one obesity-related medical condition; studies, with duration≥ 16 weeks, performed on patients with BMI>30 Kg/m^2. Remission of obstructive sleep apnea is defined as 1) C-PAP discontinuation [1], 2) symptoms remission (with specific questionnaires), or 3) Apnea-Hypopnea Index< 5 events/h at polysomnography [1]. Remission of dyslipidemia is defined as normal values of cholesterol without medications [2]. Remission for hypertension is defined as normal blood pressure values without any antihypertensive medication [3]. Complete and partial diabetes remission is defined following American Diabetes Association guidelines [4].
1.4, 2.44, 3.4	Reduction of all-cause mortality	RCT	Patients aged ≥18 years aged with obesity; studies performed on patients with BMI≥30 Kg/m^2 and duration≥ 16 weeks. Mortality at endpoint.
1.5, 2.5, 3.5	Improvement of quality of life	RCT	Patients aged ≥18 years, with obesity; studies performed on patients with BMI≥30 Kg/m^2 and duration≥ 16 weeks. Quality of life measured with either generic or disease-specific questionnaires at endpoint.
1.6, 2.6, 3.6	Perioperative surgical complications	RCT	Patients aged ≥18 years, with obesity; studies performed on patients with BMI≥30 Kg/m2 and duration≥ 16 weeks. Surgical complications during surgical procedures or hospital stay.
1.7, 2.7, 3.7	Serious adverse events (surgical and non-surgical)	RCT	Patients aged ≥18 years, with obesity ± diabetes; studies, with duration≥ 52 weeks, performed on patients with BMI ≥30Kg/m^2. Any serious adverse events during surgical procedures or during follow-up period at endpoint.

* OSAS: Obstructive Sleep Apnea Syndrome; C-PAP: Continuous-Positive Airway Pressure.

References

1. Stenberg E, Ottosson J, Näslund E. Remission of Obesity-Related Sleep Apnea and Its Effect on Mortality and Cardiovascular Events after Metabolic and Bariatric Surgery: A Propensity-Matched Cohort Study. J Am Coll Surg. 2024 Aug 1;239(2):77-84. doi: 10.1097/XCS.0000000000001047.
2. Benaiges D, Goday A, Casajoana A, Flores-Le Roux JA, Fitó M, Pozo OJ, Serra C, Pera M, Llauradó G, Climent E, Villatoro M, Lazaro I, Castañer O, Pedro-Botet J. Short-term effects of gastric bypass versus sleeve gastrectomy on high LDL cholesterol: The BASALTO randomized clinical trial. Cardiovasc Diabetol. 2024 Jun 15;23(1):205. doi: 10.1186/s12933-024-02296-x.
3. Wang L, Lin M, Yu J, Fan Z, Zhang S, Lin Y, Chen X, Peng F. The Impact of Bariatric Surgery Versus Non-Surgical Treatment on Blood Pressure: Systematic Review and Meta-Analysis. Obes Surg. 2021 Nov;31(11):4970-4984. doi: 10.1007/s11695-021-05671-9.
4. Yang Y, Miao C, Wang Y, He J. The long-term effect of bariatric/metabolic surgery versus pharmacologic therapy in type 2 diabetes mellitus patients: A systematic review and meta-analysis. Diabetes Metab Res Rev. 2024 Jul;40(5):e3830. doi: 10.1002/dmrr.3830.
